# Discovery of Flavonoids from *Scutellaria baicalensis* with Inhibitory Activity Against PCSK 9 Expression: Isolation, Synthesis and Their Biological Evaluation

**DOI:** 10.3390/molecules23020504

**Published:** 2018-02-24

**Authors:** Piseth Nhoek, Hee-Sung Chae, Jagadeesh Nagarajappa Masagalli, Karabasappa Mailar, Pisey Pel, Young-Mi Kim, Won Jun Choi, Young-Won Chin

**Affiliations:** College of Pharmacy and Integrated Research Institute for Drug Development, Dongguk University-Seoul, 32 Dongguk-lo, Ilsandong-gu, Goyang-si, Gyeonggi-do 10326, Korea; piseth2306@gmail.com (P.N.); chaeheesung83@gmail.com (H.-S.C.); mnjagadeesh123@gmail.com (J.N.M.); Kitty_1506@yahoo.com (K.M.); jully.christ07@gmail.com (P.P.); 0210121@hanmail.net (Y.-M.K.)

**Keywords:** *Scutellaria baicalensis*, flavonoid, PCSK9, SREBP-1, low density lipoprotein receptor

## Abstract

Nine flavonoids were isolated and identified from a chloroform-soluble fraction of the roots of *Scutellaria baicalensis* through a bioactivity-guided fractionation using a proprotein convertase subtilisin/kexin type 9 (PCSK9) monitoring assay in HepG2 cells. All structures were established by interpreting the corresponding spectroscopic data and comparing measured values from those in the literature. All compounds were assessed for their ability to inhibit PCSK9 mRNA expression; compounds **1** (3,7,2′-trihydroxy-5-methoxy-flavanone) and **4** (skullcapflavone II) were found to suppress PCSK9 mRNA via SREBP-1. Furthermore, compound **1** was found to increase low-density lipoprotein receptor protein expression. Also, synthesis of compound **1** as a racemic mixture form (**1a**) was completed for the first time. Natural compound **1** and synthetic racemic **1a** were evaluated for their inhibitory activities against PCSK9 mRNA expression and the results confirmed the stereochemistry of **1** was important.

## 1. Introduction

Cardiovascular disease (CVD) is a class of well-recorded diseases that lead to prominent adult mortality worldwide. High cholesterol level in the plasma has been identified as a major cause of CVD [1]. Cholesterol is an essential substance to cell membranes, but excess levels of it, either in biosynthesis, uptake, or storage, is tremendously associated to heart-related diseases. Over the past 20 years, statin therapy has been the standard treatment for successful cholesterol reduction [2]. However, some patients with familial hypercholesterolemia (FH) fail to reach the desired cholesterol concentration using common statin therapy [3,4] even at maximal statin dose. Low density lipoprotein receptors (LDL-R) are expressed on the surface of hepatocytes and bind to LDL particles [5,6]. This LDL-R/LDL complex is primarily internalized into the cell through clathrin-coated vesicles, after which the complex dissociates and LDL-C gets degraded into lipids and amino acids. After this process, LDL-R moves back to the cell surface, suggesting that the recycling of LDL-R seems to be significant in lowering LDL-C levels in the plasma [7,8]. In patients with FH, this recycling system is impaired by high concentrations of proprotein convertase subtilisin/kexin type 9 (PCSK9). PCSK9 is known to bind LDL-R and prevent its recycling, which decreases LDL-R expression on cell surfaces and consequently results in high levels of LDL-C in the plasma, leading to hypercholesterolemia [9,10]. Therefore, PCSK9 inhibition would increase LDL-R expression, which in turn decreases cholesterol levels in the plasma. Thus far, two antibody drugs have been approved as PCSK9 inhibitors, while no small molecules have ever reached at the clinical trials [11,12]. Moreover, only a few natural products in either extracts or individual chemicals have been explored for PCSK9 regulation [13,14,15,16,17,18].

*Scutellaria baicalensis* belongs to the Lamiaceae family [19] and is native to the soil of Asian countries. The plant has been used as a food additive and for traditional medicine [20]; its roots have been used for the treatment of allergies, inflammation [21,22], fever, dysentery [23], pneumonia, influenza [24], diarrhea [25], and have potential anticancer activities [26,27]. Phytochemical investigations of *S. baicalensis* roots have disclosed flavonoids, phenylethanoids and sterols as the main chemical constituents [28].

## 2. Results and Discussion

### 2.1. Inhibitory Activity against PCSK9 mRNA Expression of Root Extract from S. baicalensis

**Figure 1:**
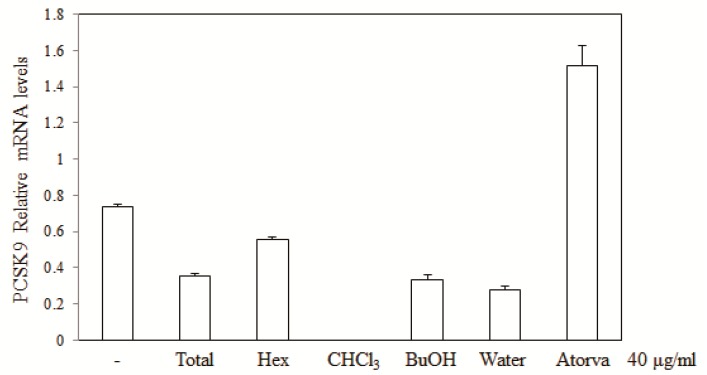
The effects of (total) methanol extracts, hexane (Hex), chloroform (CHCl_3_), butyl alcohol (BuOH) and aqueous fractions of *S.* *baicalensis* on PCSK9 mRNA expression in HepG2 cells. (Atorva = Atorvastatin).

In the preliminary screening assay to assess medicinal plant extracts for their inhibitory activity against PCSK9 mRNA expression using HepG2 cells, a methanolic extract of *S. baicalensis* was found to inhibit PCSK9 mRNA expression. Thus, subsequent partitioning with organic solvents (hexane, chloroform, butyl alcohol and water-soluble extracts) was conducted and the resultant solvent-soluble extracts were tested again using the same bioassay (Figure 1). A chloroform-soluble extract demonstrated inhibitory activities on PCSK9 mRNA expression. Therefore, the chloroform-soluble fraction was further investigated to discover potential molecules in the extract that are responsible for the inhibitory effects on PCSK9 expression.

### 2.2. Identification of Isolates ***1**–**9*** from S. baicalensis

**Figure 2:**
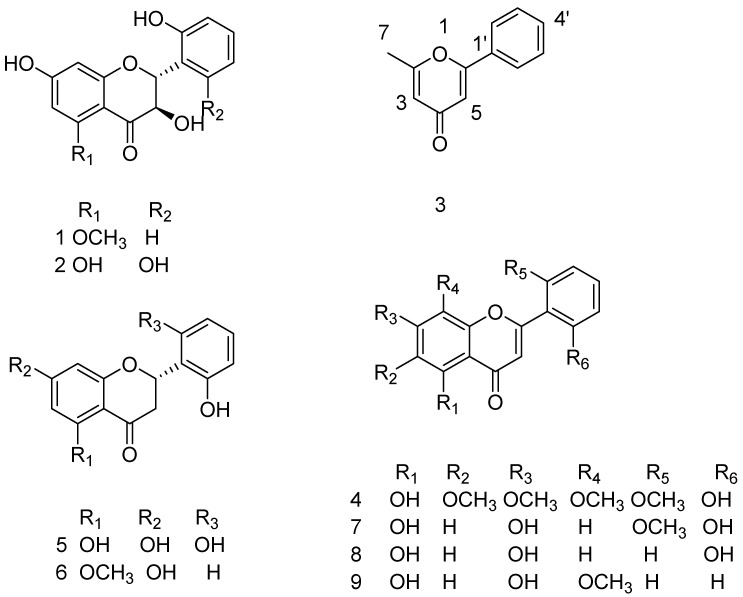
Chemical structures isolated from *S. baicalensis.*

In the present study, nine known structures were isolated (Figure 2), with structures confirmed by spectroscopic data analyses. The isolated compounds **1**–**9** were identified as follows: 3,7,2′-trihydroxy-5-methoxy-flavanone (**1**) [29], 3,5,7,2′,6′-pentahydroxyflavanone (**2**) [30], 2-methyl-6-phenylpyran-4-one (**3**) [31,32], skullcapflavone II (**4**) [33], 5,7,2′,6′-tetrahydroxyflavone (**5**) [30], 2,3-dihydro-7-hydroxy-2-(2-hydroxyphenyl)-5-methoxy-benzopyran-4-one (**6**) [34], 5,7,2′-trihydroxy-6′-methoxyflavone (**7**) [35], 5,7,2′-trihydroxyflavone (**8**) [36] and wogonin (**9**) [37,38], by extensive NMR experiments (^1^H, ^13^C, HSQC, and HMBC) and comparisons with values previously reported in the literature. Since there are no reports regarding full assignments for compound **3**, all assignments were provided in the isolation method.

### 2.3. Stereochemistry Determination of Compound ***1***

The stereochemistry of C-2 and C-3 in **1** was determined to be in a *trans* orientation based on the observation that the vicinal coupling constant between H-2 and H-3 was 11.2 Hz, consistent with what is known in the literature regarding compound **2** [39,40]. Furthermore, circular dichroism (CD) spectroscopy of **1** revealed a positive Cotton effect at 328 nm and a negative Cotton effect at 299 nm, suggesting that the configurations of C-2 and C-3 were 2*R* and 3*R*, respectively [41]. The structure of **1** was thus characterized as (2*R*,3*R*), 3,7,2′-trihydroxy-5-methoxyflavanone.

### 2.4. Inhibitory Activity against PCSK9 mRNA Expression of Isolates from S. baicalensis

**Figure 3:**
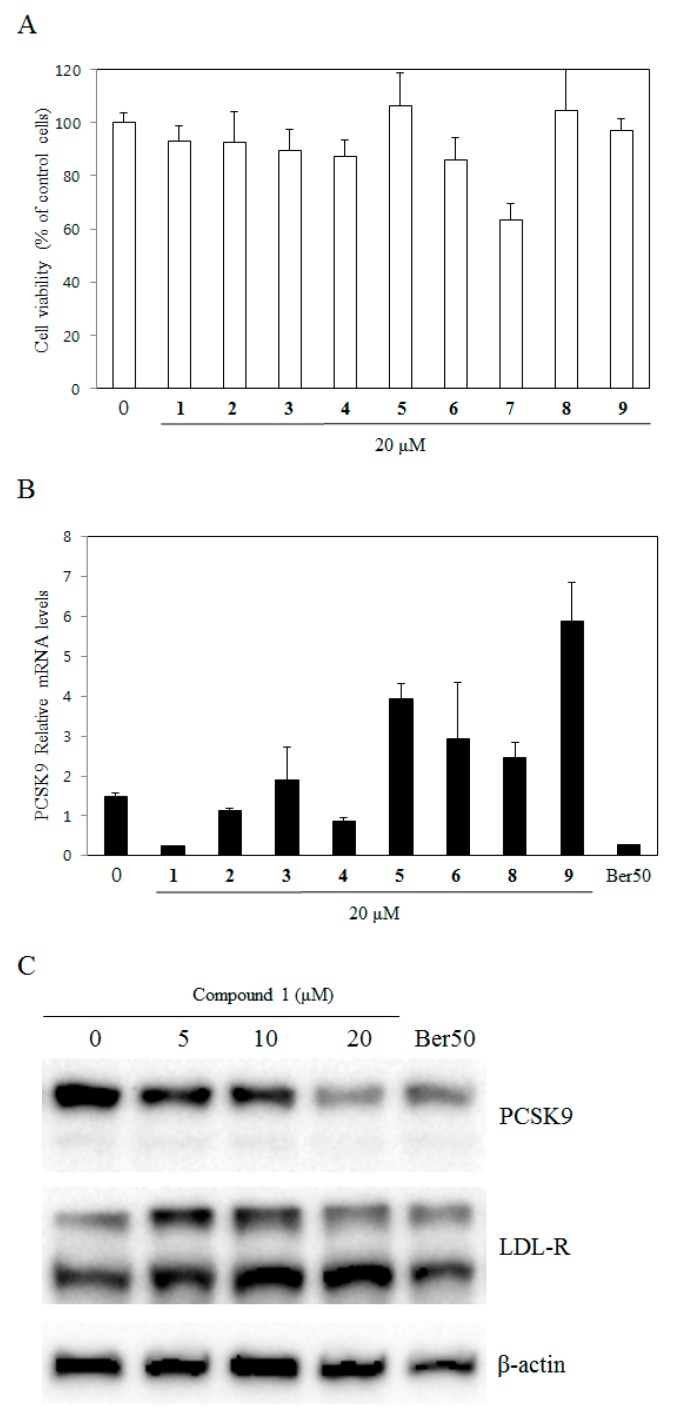
Effects of compounds from *S. baicalensis* on Cell viability, PCSK9 and LDL-R expression in HepG2 cells by MTT, qRT-PCR and western blot analysis. (**A**) Cells grown were treated with 20 μM of compounds for 24 h, and cell viability was assessed by the MTT assay; (**B**) Expression of PCSK9 was assayed by qRT-PCR in cells treated with compounds **1**–**9** except for **7**, and berberine (50 μM) for 24 h; (**C**) Expression of PCSK9 and LDL-R were assayed by western blots in cells treated with (**1**) and atorvastatin for 24 h. (Ber50, berberine 50 μM).

The isolated **1**–**6**, **8** and **9** from this study were evaluated for their PCSK9 mRNA expression in HepG2 cells. Compound **7** was excluded for cytotoxicity (Figure 3A). Only **1** and **4** (skullcapflavone II) exhibited inhibitory activity at 20 µM (84.4% and 42.4%, respectively) (Figure 3B). In addition, some compounds from the same plant that have been previously isolated in our laboratory [26] were tested in the same assay at 20 µM in order to assess structure-activity relationship (Supplementary Materials Figure S15). None of them were active in this assay system. From the structures **1** (active) and **6** (inactive), it was inferred that a hydroxyl group at C-3 position might provide the different activities. When the structure of skullcapflavone II (**4**) was compared with the similar structures of molsoflavone and alnetin in Supplementary Materials Figure S15, no conclusive result was reached due to the limited number of structures.

Out of the active compounds, compound **1** was further tested for its PCSK9 and LDL-R protein expression and it was found that compound **1** was able to inhibit PCSK9 and increase LDL-R protein expression, respectively (Figure 3C). As mentioned earlier, PCSK9 facilitates LDL-R degradation and prevents LDL-R recycling. Taking into consideration this function of PCSK9, compound **1** may potentially lower cholesterol levels by decreasing PCSK9 expression and concomitantly increasing LDL-R expression.

### 2.5. Comparison of Inhibitory Activity against PCSK9 mRNA Expression between Compound ***1*** and ***1a***

**Scheme 1:**
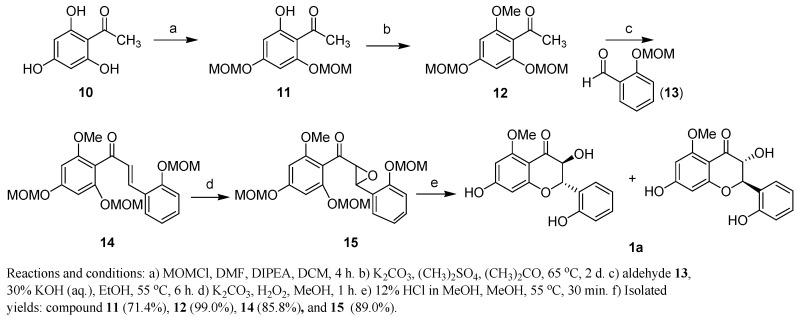
Synthesis of racemic compound **1a**.

Due to its activity, we selected compound **1** for synthesis and designed the synthetic scheme shown in Scheme 1. Our approach to synthesize racemic **1a** began with di-MOM protected 1-(2-hydroxy-4,6-bis(methoxymethyl)phenyl)ethanone (**11**), which was readily prepared from the commercially available 2′,4′,6′-trihydroxyacetophenone (**10**) by treatment with MOMCl and ethyldiisopropylamine [42]. Compound **11** was converted to chalcone **14** through methylation using dimethyl sulfate in acetone, followed by Claisen-Schmidt aldol condensation with 2-(methoxymethyl)benzaldehyde (**13**) in the presence of aqueous base [43,44]. Epoxidation of compound **14** with alkaline hydrogen peroxide at room temperature produced **15** in 89% yield [45]. Treatment of the epoxide **15** employing various acidic conditions for the purpose of instantaneous MOM deprotection and intramolecular 6-*endo* opening of epoxide afforded flavanol **1a**, however, the yield of the product was limited to 3–5%, with a variety of undesired products [44,46]. Finally, treatment of **15** with 12% conc HCl in methanol produced compound **1a** as a racemic mixture in 11% yield as shown in Scheme 1 [47].

**Figure 4:**
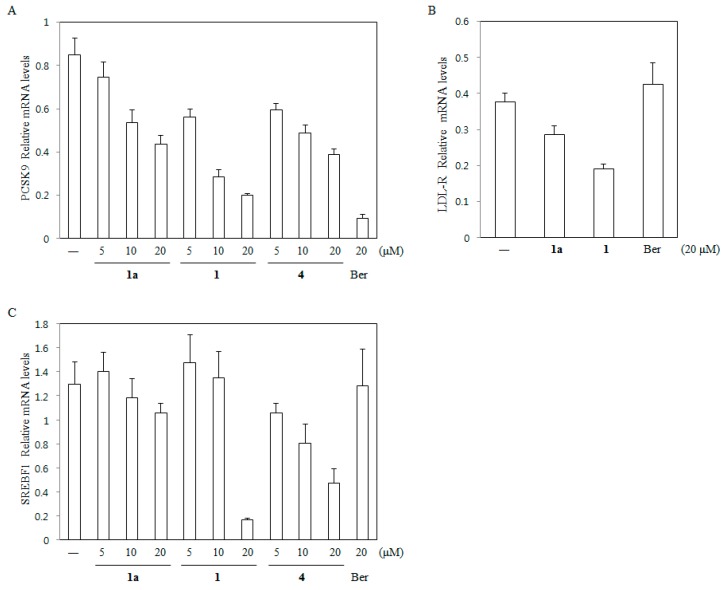
Expressions of PCSK9 (**A**) LDLR (**B**) and SREBF1 (**C**) were assayed by qRT-PCR in cells treated with compounds **1a**, **1**, **4** and Ber (berberine) at a indicated concentrations.

To compare bioactivity of natural **1** and racemic **1a**, these two compounds were tested in HepG2 cells for their inhibitory activities against PCSK9 mRNA expression. As expected (Figure 4), natural **1** seemed to be more potent than racemic **1a** because racemic **1a** are composed of two isomers. From this result, PCSK9 mRNA expression might be modulated by **1** with specific configurations.

Further analysis demonstrated that downregulation of SREBP-1 mRNA was detected in compounds **1** and **4**, suggesting inhibition of PCSK9 mRNA expression was mediated by SREBP-1 as reported in the literature [48]. Thus, it merits enantioselective synthesis of (2*R*,3*R*) compound **1** and its derivatives for further chemical modifications and in vivo studies.

## 3. Materials and Methods

### 3.1. General Information

Nuclear magnetic resonance (NMR) spectra were obtained using a Varian 400 spectrometer (Varian, Palo Alto, CA, USA) 400 MHz spectrometer operated at 400 MHz for ^1^H-NMR and at 100 MHz for ^13^C-NMR. High-resolution mass spectra data were measured on a Xevo G2 Q-TOF mass spectrometer (Waters, Milford, MA, USA). Fourier Transform Infrared (FT-IR) recorded on a Nicolet™ iS™ 5 FT-IR spectrometer (ThermoFisher Scientific, Madison, WI, USA) were used. Ultraviolet visible spectroscopy was performed using a DU 730 UV/Vis spectrophotometer (Beckman Coulter GmbH, North Rhine-Westphalia, Germany). Optical rotation and CD data are also given in the Supplementary file. Semi-preparative high performance liquid chromatography (HPLC) was performed on a system equipped with a Gilson 321 pump and Gilson 172 Diode Array Detector (Gilson, Madison, WI, USA) using YMC-pack Ph (250 × 20 mm) and YMC-pack Ph (250 × 10 mm) HPLC columns (YMC, Kyoto, Japan). Water was purified using a Milli-Q system (Waters Corporation, Milford, MA, USA). Column chromatography on C-18 RP silica gel (Cosmosil, Kyoto, Japan) and Sephadex LH-20 (GE Healthcare, Uppsala, Sweden) was conducted, and TLC analysis on silica gel 60 F_254_ plates (Merck, Darmstadt, Germany) was done. The spots were visualized by spraying with 10% aqueous H_2_SO_4_.

### 3.2. Cell Culture and Chemical Reagents

The HepG2 human hepatocellular liver cell line was obtained from the Korea Research Institute of Bioscience and Biotechnology (Daejeon, Korea) and grown in Eagle’s Minimum Essential Medium (EMEM) containing 10% fetal bovine serum and 100 U/mL penicillin/streptomycin sulfate. Cells were incubated in a humidified 5% CO_2_ atmosphere at 37 °C. EMEM, penicillin, and streptomycin were purchased from Hyclone (Logan, UT, USA). Bovine serum albumin was purchased from Sigma-Aldrich (St. Louis, MO, USA). Antibodies against PCSK9, LDL-R, and β-actin were purchased from Abcam, Inc. (Cambridge, MA, USA). PCSK9, LDL-R, SREBP1, and glyceraldehyde-3-phosphate dehydrogenase (GAPDH) oligonucleotide primers were purchased from Bioneer Corp. (Daejeon, Korea). The solvents for extraction and isolation (methanol, ethyl acetate, *n*-butyl alcohol, chloroform, *n*-hexane, etc.) were purchased from SK Chemical (Seoul, Korea). The solvents for HPLC-grade acetonitrile (MeCN) and methanol were also purchased from SK Chemical. The solvent for NMR (CD_3_OD) was obtained from Cambridge Isotope Laboratories, Inc. (Andover, MA, USA).

### 3.3. Extraction, Isolation and Synthetic Method

#### 3.3.1. Isolation Method

The roots of *S. baicalensis* (3 kg) was extracted with MeOH three times (18 L × 3) at room temperature and evaporated in vacuo. The concentrated MeOH extract (558.1 g) was suspended in H_2_O and successively partitioned between hexane, chloroform, and butyl alcohol, to give the inactive residues of hexane-soluble fraction, butyl alcohol-soluble fraction, and water-soluble fraction, and the active residue of chloroform-soluble fraction (34.7 g). The chloroform-soluble fraction (33.8 g, SBC) was chromatographed over a silica gel column chromatography (CC) (5 × 90 cm, 685 g) using a gradient of increasing polarity with chloroform:MeOH (200:0–1:1) as eluted solvents system and was fractioned into 50 sub-fractions (SBC1~50). SBC29 (8.8 g) was subjected to medium pressure liquid chromatography (MPLC), carried out with the binary system of MeOH–H_2_O (0:100, 100:0) to give 8 sub-fractions (SBC 29-1~8). SBC 29-6 was purified by size exclusion chromatography on Sephadex LH-20 CC by using chloroform:MeOH (1:1) as eluent, and washing by acetone to give 11 sub-fractions (SBC 29-6A~K), including compound **4** (227.2 mg). SBC 29-7 was taken out 100 mg to subject to semi-preparative high performance liquid chromatography (HPLC) performed on a 250 × 21.2 i.d.mm, Acclaim Polar Advantage 2, Thermo Scientific column (5 µm, Life Technologies Korea LCC, Seoul, Korea), using MeCN:H_2_O (45:65) as solvent system, flow rate 3 mL/min by isocratic elution method for 30 min and MeCN 100% for 5 min to afford compound **9** (*t*_R_ 17.3 min, 1.3 mg). Sub-fraction SBC 44 and 45 were mixed together and separated on Sephadex LH-20 column with chloroform:MeOH (1:1), to give 14 sub-fractions (SBC 44A~N), includes compound **5** (10.3 mg). Sub-fraction SBC 44-J was subjected to HPLC separation (250 × 20 i.d.mm, YMC-Pack Pro C18 RS, 5 µm), using MeCN: H_2_O as eluted solvent, flow rate 5 mL/min, by isocratic elution, using MeCN 30% for 25 min, then increase directly to MeCN 35% and then maintained in the same isocratic mode for 15 min and MeCN 100% for 5 min to give compound **1** (*t*_R_ 23.2 min, 2.4 mg). SBC 44-I was subjected to HPLC separation (250 × 21.2 i.d.mm, Acclaim Polar Advantage 2, 5 µm) using MeCN:H_2_O, flow rate 3 mL/min, eluted with MeCN 30% for 25 min, then MeCN 35% for 15 min and MeCN 100% for 5 min to give compound **6** (*t*_R_ 29.7 min, 1 mg), compound **7** (*t*_R_ 41.7 min, 2.4 mg) and compound **8** (*t*_R_ 43.8 min, 1 mg). Sub-fraction SBC 42 was combined with sub-fraction SBC 41 and purified on Sephadex LH-20 CC to give 10 sub-fractions (SBC 42A~J). Sub-fraction SBC 42-I was further purified by HPLC (250 × 21.2 i.d.mm, Acclaim Polar Advantage 2, 5 µm), with MeCN:H_2_O (30:70), flow rate 3 mL/min by isocratic mode for 30 min and MeCN 100% for 5 min to afford compound **3** (*t*_R_ 22.5 min, 3.2 mg). Sub-fraction SBC 48 was mixed with sub-fraction SBC 46 and 47 and separated on Sephadex LH-20 CC by using chloroform: MeOH (1:1) solvent system and washing by acetone 100% to afford 8 sub-fractions (SBC 48A~H). Compound **2** (*t*_R_ 12.5 min, 1.6 mg) was isolated from sub-fraction SBC48-G by HPLC separation (250 × 21.2 i.d.mm, Acclaim Polar Advantage 2, 5 µm) with MeCN:H_2_O (25:75), flow rate 3 mL/min by isocratic elution mode for 20 min and MeCN 100% for 5 min.

*2-Methyl-6-phenyl-4H-pyran-4-one* (**3**): ^1^H-NMR (400 MHz, CD_3_OD) δ: 6.30 (1H, d, *J* = 2.0 Hz, H-3), 6.82 (1H, *d*, *J* = 2.0 Hz, H-5), 7.55 (3H, m, H-3′, H-4′, H-5′), 7.90 (2H, dd, *J* = 4.5, 1.6 Hz, H-2′, H6′), 2.43 (3H, s, H-7). ^13^C-NMR (100 MHz, CD_3_OD) δ 169.2 (C-2), 114.4 (C-3), 183.0 (C-4), 110.7 (C-5), 166.3 (C-6), 19.7 (C-7), 132.2 (C-1′), 127.1 (C-2′, C-6′), 130.2 (C-3′, C-4′, C-5′).

#### 3.3.2. Synthesis Method

Phloroacetophenoe monohydrate (>98% purity) and hydrogen peroxide (35% in water) were purchased from TCI Co., Ltd., (Tapei, Taiwan). Salicylaldehyde (97%) was purchased from Junsei Chemicals Co., Ltd. (Chuo-ku, Tokyo, Japan). Methoxymethyl chloride (>95%) was purchased from Kanto Chemicals (Chuo-ku, Tokyo, Japan) with yield. Potassium carbonate (99.5%) was purchased from Samchun Chemicals (Gangnam-gu, Seoul, South Korea). Terahydrofuran (>99.9%) was purchased from Sigma-Aldrich (St. Louis, MO, USA). N-Ethyldiisopropylamine (99%) was purchased from Alfa Aesar (Gangnam-gu, Seoul, South Korea).

*1-(2-Hydroxy-4,6-bis(methoxymethyl)phenyl)ethanone* (**11**). To a stirred solution of 1-(2,4,6-trihydroxyphenyl)ethanone (**10**, 1 g, 5.9 mmol) in CH_2_Cl_2_ (36 mL), was added DMF (1.2 mL). The reaction mixture was cooled to 0 °C, DIPEA (3.1 mL, 17.7 mmol) was added and stirred for 5 min. Chloro(methoxy)methane (1 g, 12.4 mmol) in CH_2_Cl_2_ (5.8 mL) was next added dropwise to the reaction mixture under a N_2_ atmosphere. The mixture was warmed to room temperature and stirred for 4 h. The reaction mass was quenched with sat. aq. NH_4_Cl, and the organic layer was separated. The aqueous layer was further extracted with CH_2_Cl_2_ (2 × 50 mL), the combined organic layer was washed with brine solution and dried over MgSO_4_, filtered, and evaporated under vacuum. The residue was purified by column chromatography (petroleum ether/ethyl acetate, 8:2, *v*/*v*) to afford compound **11** as a colorless oil (0.95 g, 71.4%). ^1^H-NMR (CDCl_3_) δ 13.74 (s, 1H), 6.26 (s, 1H), 6.24 (s, 1H), 5.26 (s, 2H), 5.17 (s, 2H), 3.52 (s, 3H), 3.47 (s, 3H), 2.66 (s, 3H). ^13^C-NMR (CDCl_3_) δ 203.2, 166.8, 163.5, 160.4, 106.9, 97.1, 94.5, 94.0, 56.7, 56.5, 33.0.

*1-(2-Methoxy-4,6-bis(methoxymethyl)phenyl)ethanone* (**12**). To a stirred solution of compound **11** (0.95 g, 3.7 mmol) in acetone (20 mL), K_2_CO_3_ (1.5 g, 11.1 mmol) and (CH_3_)_2_SO_4_ (2 × 0.26 mL, 5.5 mmol) were added at 15 min intervals at room temperature. The reaction mixture was heated to reflux and maintained for 2 d. The reaction mass was cooled to room temperature, filtered and the filtrate was evaporated to dryness. The crude product was purified by column chromatography (petroleum ether/ethyl acetate, 8:2, *v*/*v*) to afford compound **12** as a pale brown oil (1 g, 99.0%). ^1^H-NMR (CDCl_3_) δ 6.45 (*d*, *J* = 2.0 Hz, 1H), 6.31 (*d*, *J* = 2.0 Hz, 1H), 5.16 (s, 2H), 5.14 (s, 2H), 3.78 (s, 3H), 3.48 (s, 3H), 3.46 (s, 3H), 2.48 (s, 3H). ^13^C-NMR (CDCl_3_) δ 201.8, 159.7, 157.9, 155.4, 115.8, 95.8, 94.8, 94.4, 93.8, 56.3, 56.2, 55.8, 32.5.

*(E)-1-(2-Methoxy-4,6-bis(methoxymethyl)phenyl)-3-(2-(methoxymethyl)phenyl)prop-2-en-1-one* (**14**). To a stirred solution of compound **12** (0.45 g, 1.66 mmol) in ethanol (10 mL) at 0 °C, was added aq. KOH (4.5 mL, 30% solution) dropwise. After stirring for 10 min then 2-(methoxymethyl) benzaldehyde (**13**, 0.415 g, 2.49 mmol) was added in one portion. The reaction mass was heated to 55 °C and maintained for 6 h. The reaction progress was monitored by TLC. The reaction mass was cooled to 0 °C, acidified to pH 5–6 using 2 N HCl, and extracted with CH_2_Cl_2_ (3 × 25 mL). The combined organic layer was washed with brine solution, dried over MgSO_4_, filtered and evaporated under vacuum. The crude product was purified by column chromatography (petroleum ether/ethyl acetate, 7:3, *v*/*v*) to afford compound **14** as a low melting solid (0.6 g, 85.8%**)**. ^1^H-NMR (CDCl_3_) δ 7.75 (*d*, *J* = 16 Hz, 1H), 7.56 (*d*, *J* = 7.6, 1.2 Hz, 1H), 7.31 (t, *J* = 8.8, 1.6, Hz, 1H), 7.12 (*d*, *J* = 8.0 Hz, 1H), 7.04 (*d*, *J* = 16.4 Hz, 1H), 7.00 (t, *J* = 7.6 Hz, 1H), 6.51 (*d*, *J* = 2.0 Hz, 1H), 6.36 (*d*, *J* = 2.0 Hz, 1H), 5.20 (s, 2H), 5.19 (s, 2H), 5.11 (s, 2H), 3.76 (s, 3H), 3.50 (s, 3H), 3.44 (s, 3H), 3.40 (s, 3H). ^13^C-NMR (CDCl_3_) δ 194.6, 159.8, 158.5, 156,2, 156.0, 139.8, 131.5, 129.5, 128.6, 124.7, 122.0, 115.0, 114.0, 95.9, 94.7, 94.6, 94.5, 94.0, 56.2, 55.9.

*(2-Methoxy-4,6-bis(methoxymethyl)phenyl)(3-(2-(methoxymethyl)phenyl)oxiran-2-yl)methanone* (**15**). To a stirred solution of compound **14** (0.07 g, 0.18 mmol) in methanol (6 mL), was added K_2_CO_3_ (0.069 g, 0.5 mmol) followed by dropwise addition of H_2_O_2_ (0.13 mL, 1.3 mmol, 35% aq. solution) at room temperature. The reaction mass was stirred for 1 h. After completion of starting material, mixture was diluted with ether and washed twice with saturated aq. NH_4_Cl solution. The combined organic layer was dried over MgSO_4_, filtered and evaporated under vacuum. The crude product was purified by column chromatography (petroleum ether/ethyl acetate, 7:3, *v*/*v*) to afford compound **15** as a light green oil (0.065 g, 89.0%). ^1^H-NMR (CDCl_3_) δ 7.25 (m, 2H), 7.19 (*d*, *J* = 8.0, 2.0, Hz, 1H), 7.09 (*d*, *J* = 8.0 Hz, 1H), 6.99 (t, *J* = 8.0 Hz, 1H), 6.45 (*d*, *J* = 2.0 Hz, 1H), 6.31 (*d*, *J* = 2.0 Hz, 1H), 7.39 (*d*, *J* = 2.0 Hz, 1H), 3.87 (*d*, *J* = 2.0 Hz, 1H), 3.77 (s, 3H), 3.47 (s, 3H), 3.45 (s, 3H), 3.38 (s, 3H), ^13^C-NMR (CDCl_3_) δ 196.8, 161.0, 159.4, 157.1, 155.9, 129.4, 125.4, 125.3, 122.0, 114.1, 111.7, 95.7, 94.8, 94.6, 94.3, 93.7, 63.7, 56.3, 56.3, 56.2, 55.9, 55.0.

*2,3-Dihydro-3,7-dihydroxy-2-(2-hydroxyphenyl)-5-methoxychromen-4-one* (**1a**). To a stirred solution of compound **15** (0.05 g, 0.11 mmol) in MeOH (2 mL), was added HCl in methanol (0.2 mL, 12% Wt) at room temperature. The reaction mass was heated to 55 °C and stirred for 30 min. After cooling, the reaction mixture was concentrated in vacuo, the crude product was then purified by prep. TLC (silica gel, CH_2_Cl_2_/MeOH, 9.5:0.5, *v*/*v*) to yield **1a** (4 mg, 11.7%) as a white solid of m.p. 124–126 °C. ^1^H-NMR (CD_3_OD) δ 7.41 (1H, *dd*, *J* =7.6, 1.5 Hz), 7.17 (1H, td, *J* =7.6, 1.5 Hz), 6.82 (1H, *d*, *J* =8.5 Hz), 6.88 (1H, *d*, *J* =7.6 Hz), 6.09 (*d*, *J* = 2.0 Hz, 1H), 5.97 (*d*, *J* = 2.0 Hz, 1H), 5.48 (1H, *d*, *J* = 11.2 Hz), 4.61 (1H, *d*, *J* = 11.2 Hz), 3.83 (3H, s). ^13^C-NMR (CD_3_OD) δ 192.2, 166.6, 165.7, 164.0, 156.4, 130.3, 128.8, 123.6, 119.8, 115.9, 102.9, 96.2, 93.5, 78.3, 72.7, 55.5. HRESIMS m/z [M + H]^+^ 303.0869 (calcd for C_16_H_14_O_6_ 303.0869).

## Supplementary Materials

Supplementary materials are available online.
