# Supplementary material for: Discovery of Flavonoids from Scutellaria baicalensis with Inhibitory Activity Against PCSK 9 Expression: Isolation, Synthesis and Their Biological Evaluation
Source: Molecules. 2018 Feb 24;23(2):504. doi: 10.3390/molecules23020504 (PMC6100156; doi:10.3390/molecules23020504)
Supplement: Supplementary file 1 [file molecules-23-00504-s001.pdf]

Supplementary Information

Discovery of flavonoids from *Scutellaria baicalensis* with inhibitory activity against PCSK 9 expression: Isolation, synthesis and their biological evaluation

Piseth Nhoek[#], Hee-Sung Chae[#], Jagadeesh Nagarajappa Masagalli, Karabasappa Mailar, Pisey Pel, Young-Mi Kim, Won Jun Choi*, Young-Won Chin*

College of Pharmacy and Integrated Research Institute for Drug Development, Dongguk University-Seoul, 32, Dongguk lo, Goyang-si, Gyeonggi-do 10326, Korea

Corresponding authors. Tel. +82-31-961-5218

E-mail address: mp89@dongguk.edu, f2744@dongguk.edu

Figure legends

Figure 1. CD and UV spectrum of compound **1** --1

Figure 2. Mass spectrum of compound **1** --2

Figure 3. IR spectrum of compound **1** --3

Figure 4. ^{1}H-NMR (400Hz, CD_3OD) spectrum of compound **1**------------------------4

Figure 5. ^{13}C-NMR (100Hz, CD_3OD) spectrum of compound **1** -----------------------5

Figure 6. ^{1}H-^{13}C HSQC spectrum of compound **1** ---------------------------------------6

Figure 7. ^{1}H-^{13}C HMBC spectrum of compound **1** ---------------------------------------7

Figure 8. ^{1}H-^{13}C-HMBC spectrum of compound **1**(correlations of 2 and H3) ---------8

Figure 9. ^{1}H-^{13}C-HMBC spectrum of compound **1**(confirmation of location of H6 and H8 and methoxyl group)---9

Figure 10. NOE difference spectrum for H6 irradiation of compound **1**--------------10

Figure 11. NOE difference spectrum for H8 irradiation of compound **1** -------------11

Figure 12. ^{1}H-NMR (400Hz, CD_3OD) spectrum of compound **1a**---------------------12

Figure 13. ^{13}C-NMR (100Hz, CD_3OD) spectrum of compound **1a** --------------------13

Figure 14. Mass spectrum of compound **1a** --14

Figure 15. Structures of isolates from *S. baicalensis* in the same laboratory ---------16

Figure 16. ^{1}H-NMR (400Hz, CD_3OD) spectrum of compound **2**-----------------------17

Figure 17. ^{13}C-NMR (100Hz, CD_3OD) spectrum of compound **2**----------------------18

Figure 18. ^{1}H-NMR (400Hz, CD_3OD) spectrum of compound **3**-----------------------19

Figure 19. ^{13}C-NMR (100Hz, CD_3OD) spectrum of compound **3**----------------------20

Figure 20. ^{1}H-NMR (400Hz, CD_3OD) spectrum of compound **4**-----------------------21
Figure 21. ^{13}C-NMR (100Hz, CD_3OD) spectrum of compound **4**----------------------22
Figure 22. ^{1}H-NMR (400Hz, CD_3OD) spectrum of compound **5**-----------------------23
Figure 23. ^{13}C-NMR (100Hz, CD_3OD) spectrum of compound **5**----------------------24
Figure 24. ^{1}H-NMR (400Hz, CD_3OD) spectrum of compound **6**-----------------------25
Figure 25. ^{13}C-NMR (100Hz, CD_3OD) spectrum of compound **6**----------------------26
Figure 26. ^{1}H-NMR (400Hz, CD_3OD) spectrum of compound **7**-----------------------27
Figure 27. ^{13}C-NMR (100Hz, CD_3OD) spectrum of compound **7**----------------------28
Figure 28. ^{1}H-NMR (400Hz, CD_3OD) spectrum of compound **8**-----------------------29
Figure 29. ^{13}C-NMR (100Hz, CD_3OD) spectrum of compound **8**----------------------30
Figure 30. ^{1}H-NMR (400Hz, CD_3OD) spectrum of compound **9**-----------------------31
Figure 31. ^{13}C-NMR (100Hz, CD_3OD) spectrum of compound **9**----------------------32
Figure 32. ^{1}H-NMR (400Hz, $CDCl_3$) spectrum of compound **11**-----------------------33
Figure 33. ^{13}C-NMR (100Hz, $CDCl_3$) spectrum of compound **11**----------------------34
Figure 34. ^{1}H-NMR (400Hz, $CDCl_3$) spectrum of compound **12**-----------------------35
Figure 35. ^{13}C-NMR (100Hz, $CDCl_3$) spectrum of compound **12**----------------------36
Figure 36. ^{1}H-NMR (400Hz, $CDCl_3$) spectrum of compound **14**-----------------------37
Figure 37. ^{13}C-NMR (100Hz, $CDCl_3$) spectrum of compound **14**----------------------38
Figure 38. ^{1}H-NMR (400Hz, $CDCl_3$) spectrum of compound **15**-----------------------39
Figure 39. ^{13}C-NMR (100Hz, $CDCl_3$) spectrum of compound **15**----------------------40
Figure 40. Isolation scheme of *S.baicalensis*---41

List of Table

Table 1. ^{1}H-and^{13}C-NMR spectroscopic (in CD_3OD) of Compound **1** and **1a**--------15

Compound **1**

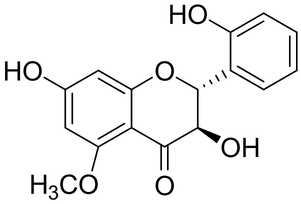

- Appearance: yellow powder
- $[\alpha]_D^{20}$ 95.11° (*c* 0.31, CD_3OD)
- UV λ_{max}^{MeOH}(log ε): 284.5 (4.55)
- HRESIMS *m/z* $[M+H]^+$ 303.0856 (calcd for $C_{16}H_{15}O_6$ 303.0990)

CD [MeOH, nm (Δε)] : 276 (-0.39), 299 (-0.13), 328 (+0.21).

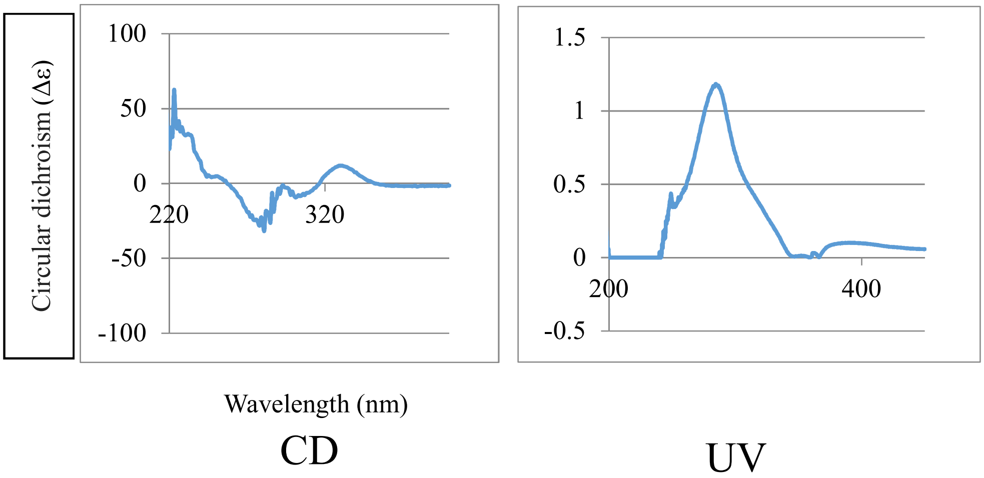

Fig. 1 CD and UV spectrum of compound **1**

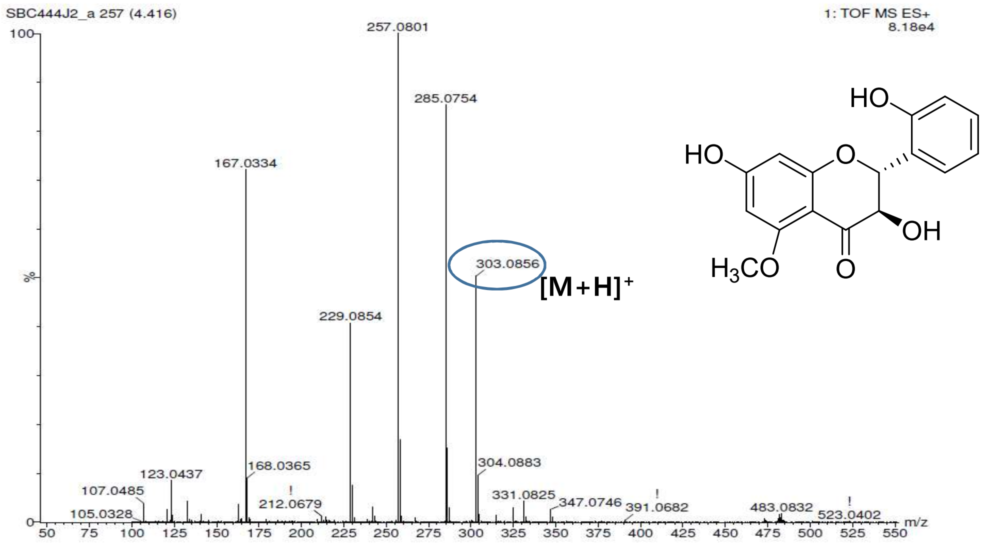

Elemental Composition Report **Page 1**

Single Mass Analysis

Tolerance = 100.0 PPM / DBE: min = -1.5, max = 50.0
Element prediction: Off
Number of isotope peaks used for i-FIT = 3

Monoisotopic Mass, Even Electron Ions
57 formula(e) evaluated with 8 results within limits (up to 100 closest results for each mass)
Elements Used:
C: 0-100 H: 0-100 O: 0-50
SBC444J2_a 257 (4.416)
1: TOF MS ES+

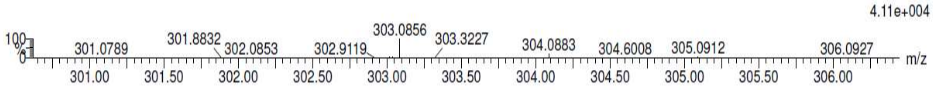

Mass	Calc. Mass	mDa	PPM	DBE	i-FIT	Norm	Conf(%)	Formula
Minimum:				-1.5				
Maximum:		5.0	100.0	50.0				
303.0856	303.0869	-1.3	-4.3	9.5	187.4	0.147	86.32	C16 H15 O6
	303.0810	4.6	15.2	18.5	193.7	6.387	0.17	C23 H11 O
	303.0927	-7.1	-23.4	0.5	197.2	9.875	0.01	C9 H19 O11
	303.0716	14.0	46.2	5.5	194.5	7.242	0.07	C12 H15 O9
	303.1021	-16.5	-54.4	13.5	190.7	3.469	3.12	C20 H15 O3
	303.0657	19.9	65.7	14.5	189.6	2.282	10.20	C19 H11 O4
	303.1080	-22.4	-73.9	4.5	194.0	6.765	0.12	C13 H19 O8
	303.0564	29.2	96.3	1.5	198.5	11.253	0.00	C8 H15 O12

Fig. 2 Mass spectrum of compound **1**

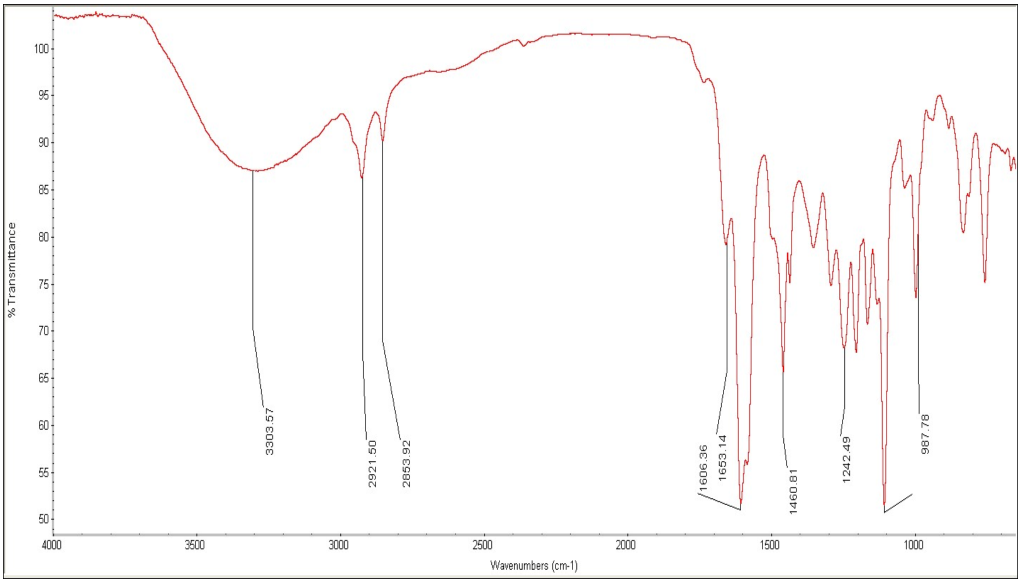

Fig. 3 IR spectrum of compound **1**

The IR spectrum of compound **1** was recorded by Attenuated Total Reflection (ATR) method in CD_3OD.

Band assignment: IR spectrum of **1** exhibited typical absorption bands at 3303 cm^{-1} due to the stretching vibration of hydroxyl group. The band at 1653 cm^{-1} can be attributed to the stretching vibration of carbonyl group.

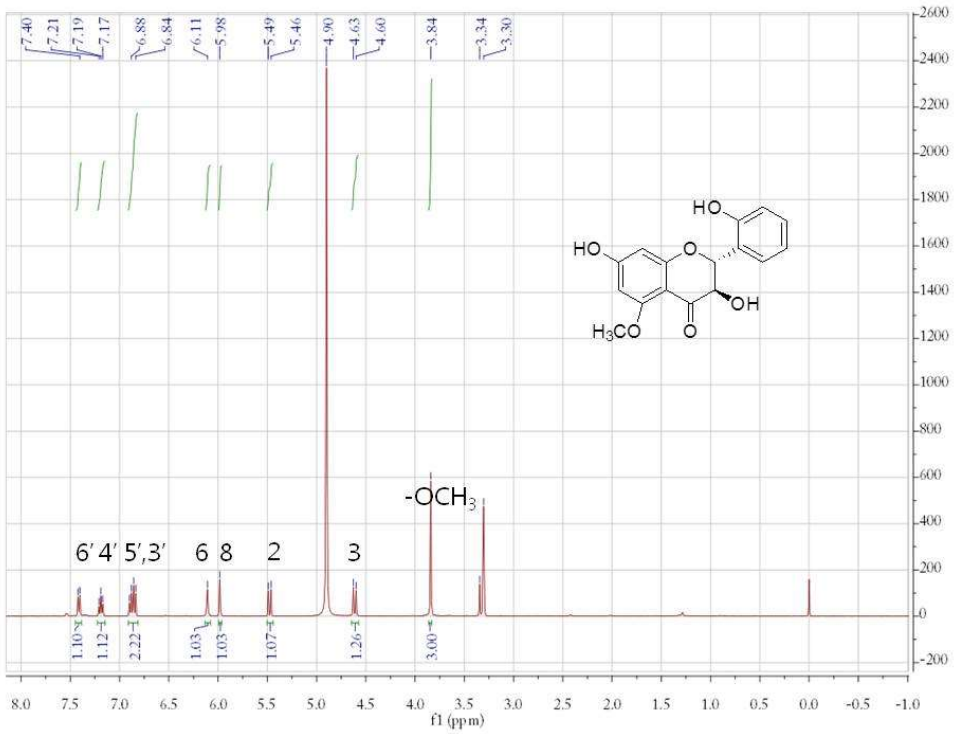

Fig. 4 ^{1}H-NMR (400 MHz, CD_3OD) spectrum of compound **1**

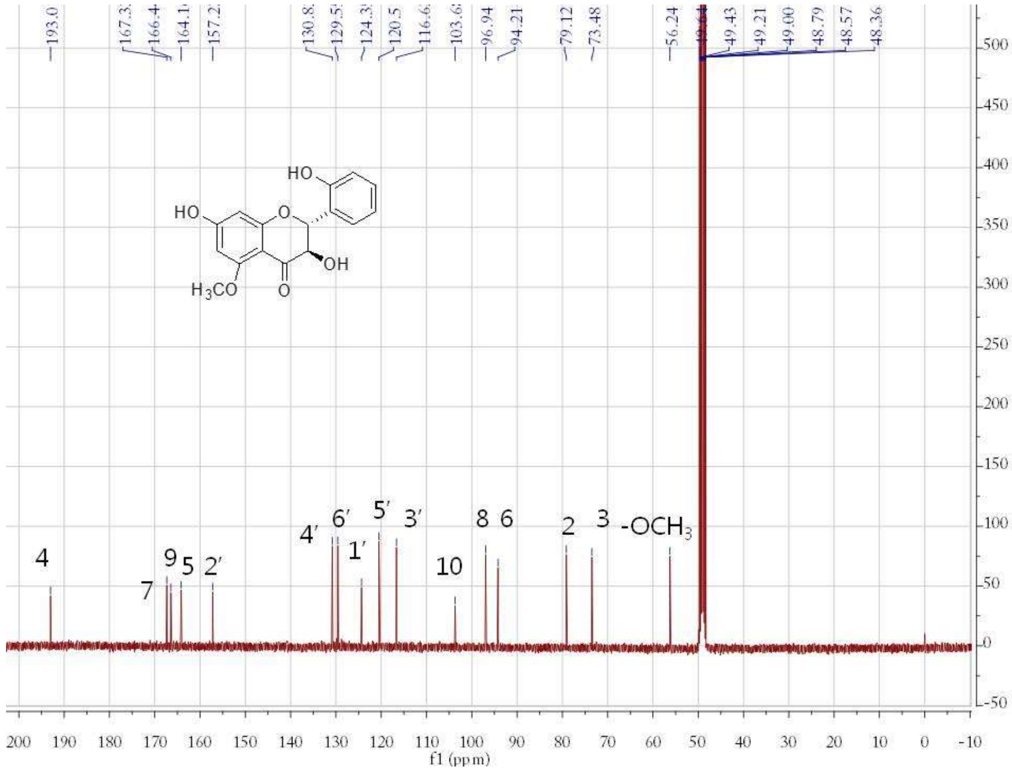

Fig.5 ^{13}C-NMR (100 MHz, CD_3OD) spectrum of compound **1**

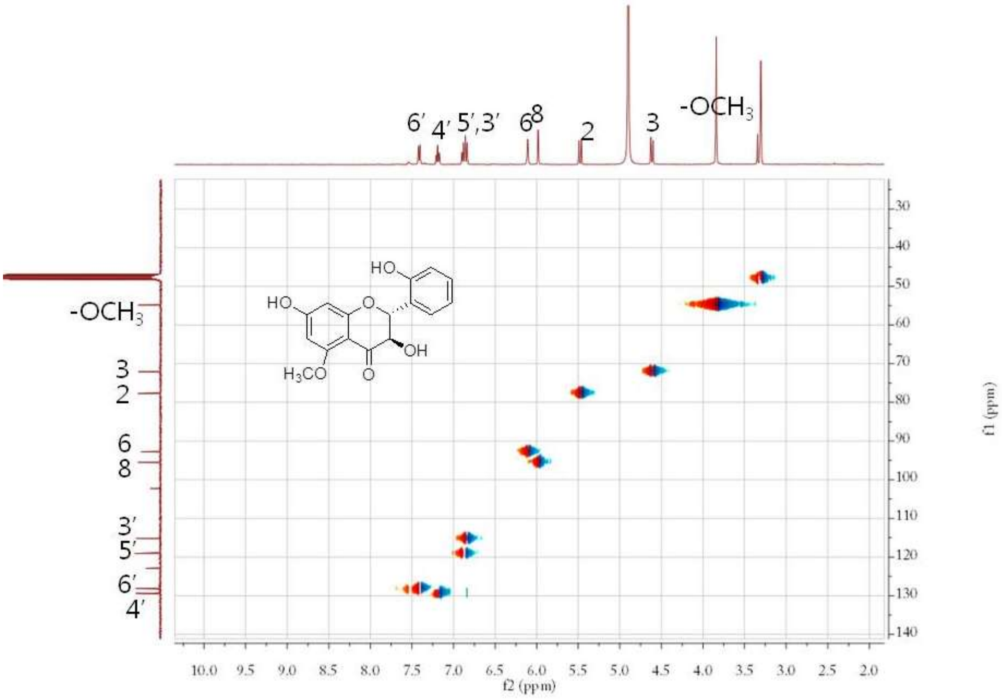

Fig. 6 ^{1}H-^{13}C-HSQC spectrum of compound **1**

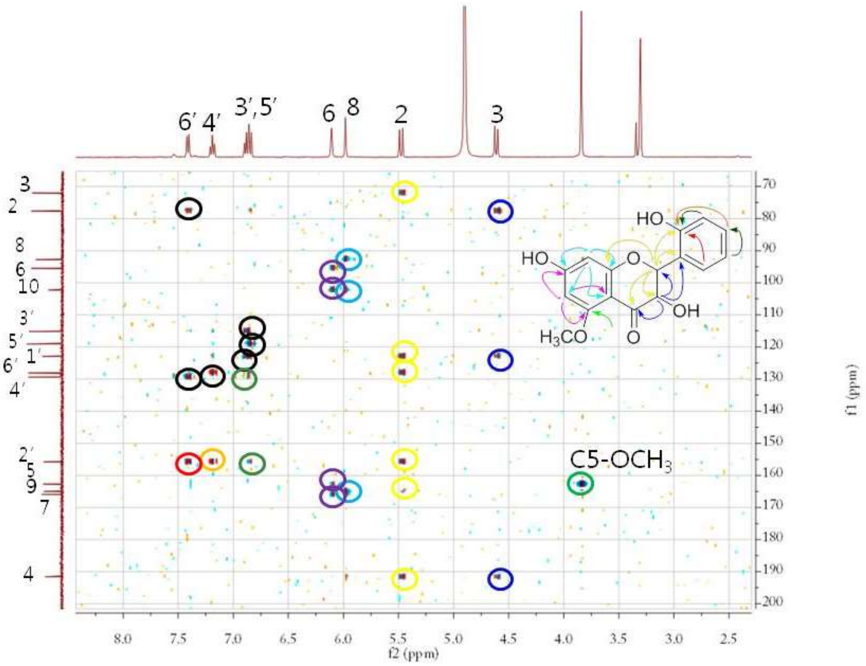

Fig. 7 ^{1}H-^{13}C-HMBC spectrum of compound **1**

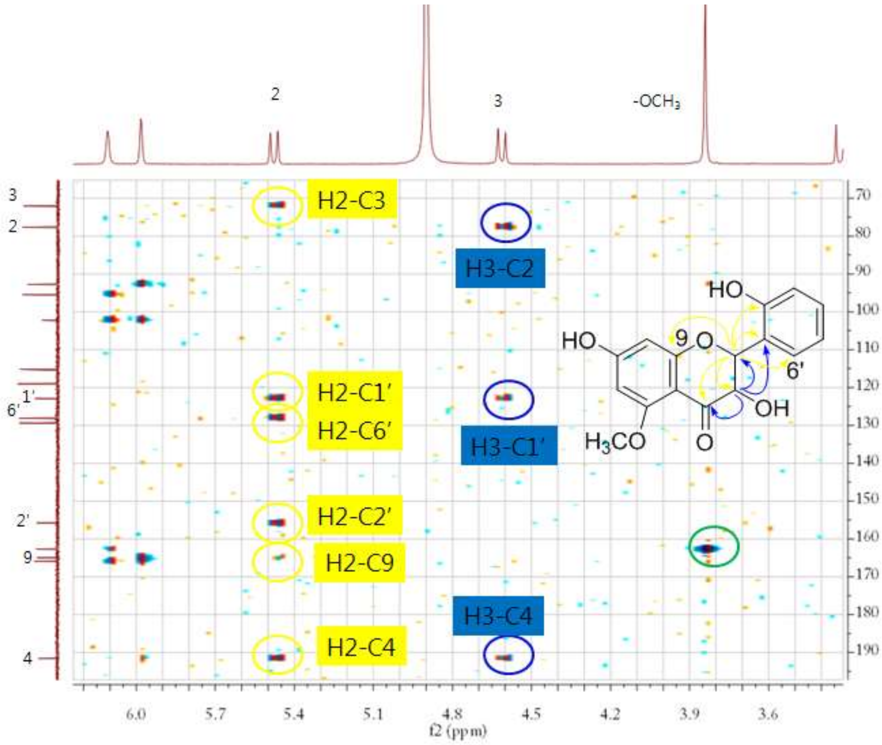

Fig. 8 ^{1}H-^{13}C-HMBC spectrum of compound **1** (confirmation of correlations of H2 and H3)

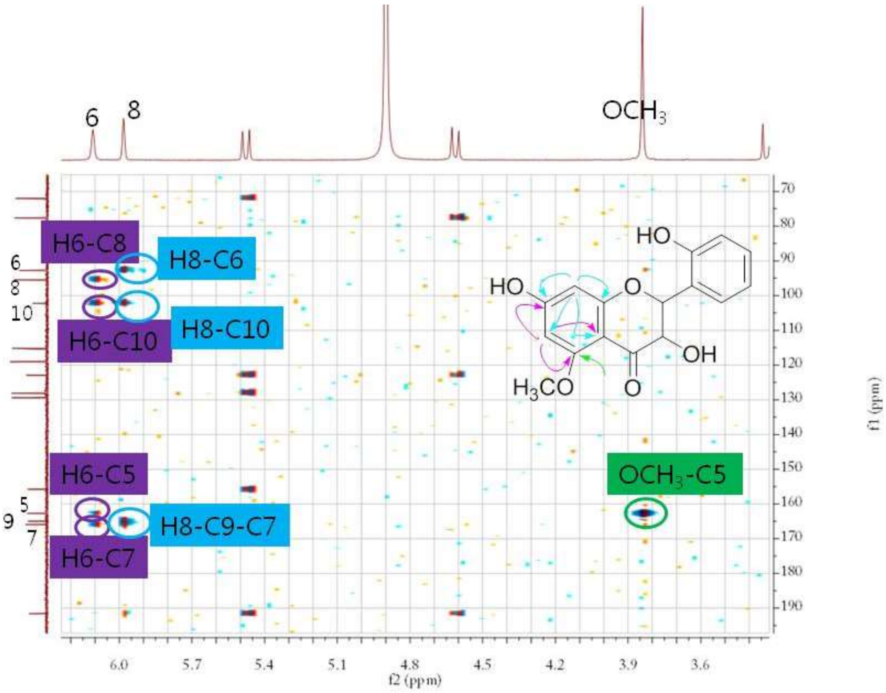

Fig. 9 ^{1}H-^{13}C-HMBC spectrum of compound **1** (confirmation of correlations of H6 and H8 and methoxyl group)

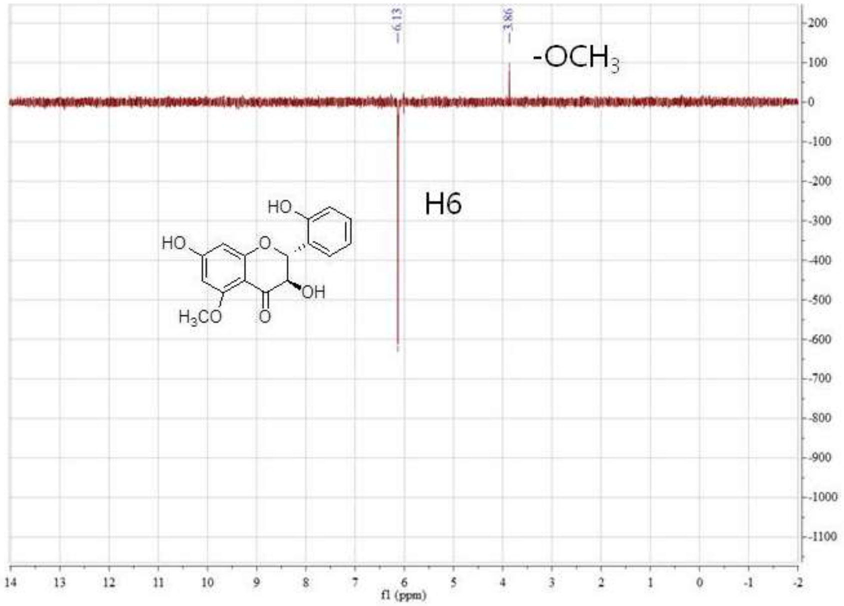

Fig. 10 NOESY difference spectrum for H6 irradiation of compound **1**.

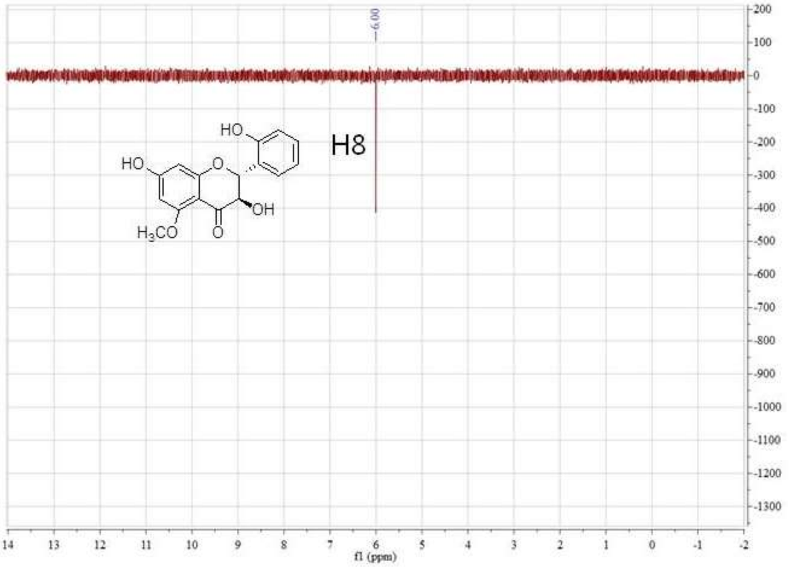

Fig. 11 NOE difference spectrum for H8 irradiation of compound **1**.

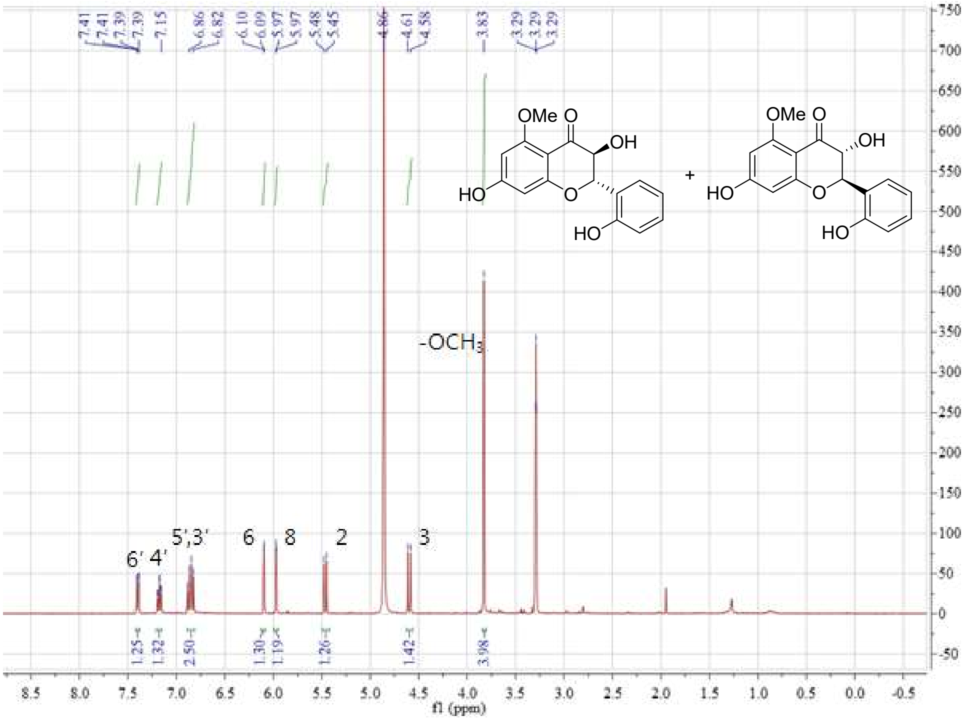

Fig. 12 ^{1}H-NMR (400Hz, CD_3OD) spectrum of compound **1a**

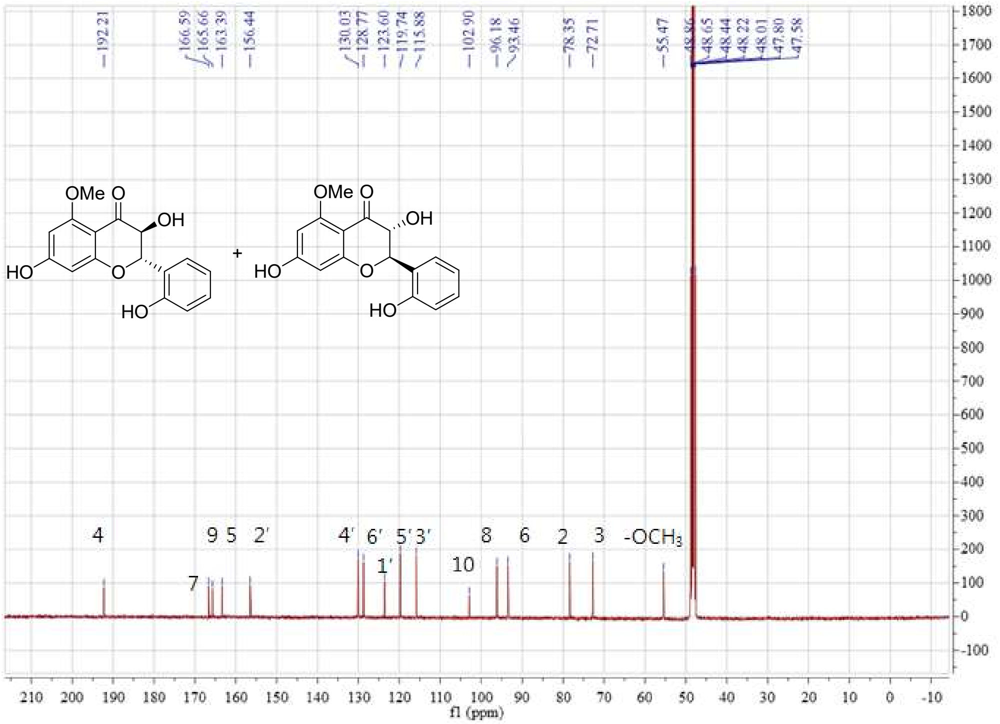

Fig. 13 ^{13}C-NMR (100Hz, CD_3OD) spectrum of compound **1a**

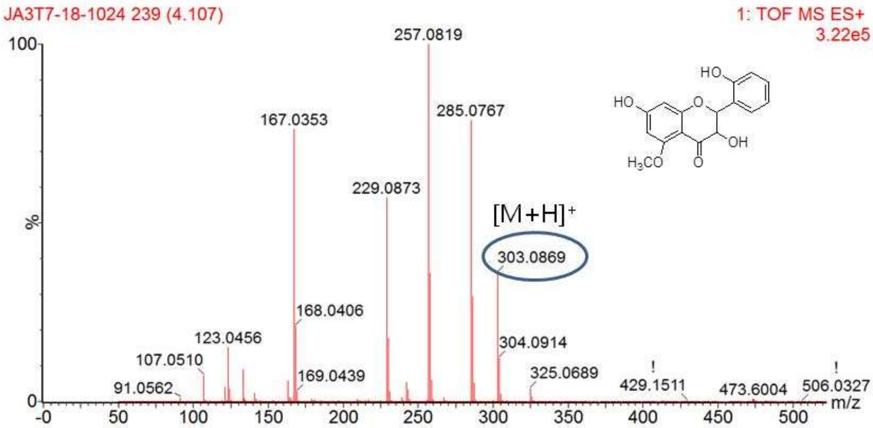

Elemental Composition Report **Page 1**

Single Mass Analysis
Tolerance = 100.0 PPM / DBE: min = -1.5, max = 50.0
Element prediction: Off
Number of isotope peaks used for i-FIT = 3

Monoisotopic Mass, Even Electron Ions
57 formula(e) evaluated with 7 results within limits (up to 100 closest results for each mass)
Elements Used:
C: 0-100 H: 0-100 O: 0-50
JA3T7-18-1024 239 (4.107)
1: TOF MS ES+

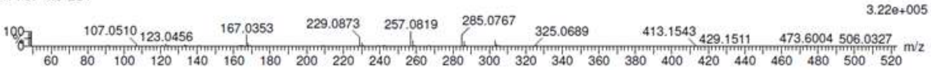

Mass	Calc. Mass	mDa	PPM	DBE	i-FIT	Norm	Conf(%)	Formula
Minimum:				-1.5				
Maximum:		5.0	100.0	50.0				
303.0869	303.0869	0.0	0.0	9.5	458.3	3.849	2.13	C16 H15 O6
	303.0927	-5.8	-19.1	0.5	461.6	7.124	0.08	C9 H19 O11
	303.0810	5.9	19.5	18.5	454.7	0.238	78.80	C23 H11 O
	303.1021	-15.2	-50.2	13.5	456.6	2.203	11.05	C20 H15 O3
	303.0716	15.3	50.5	5.5	460.3	5.842	0.29	C12 H15 O9
	303.1080	-21.1	-69.6	4.5	460.3	5.864	0.28	C13 H19 O8
	303.0657	21.2	69.9	14.5	457.0	2.608	7.37	C19 H11 O4

Fig. 14 Mass spectrum of compound **1a**

Table of ^{1}H-and^{13}C-NMR spectroscopic (in CD_3OD) of Compound **1** and **1a**

	1		1a	
No.	δ_H *a)*	δ_C *b)*	δ_H *a)*	δ_C *b)*
1	-	-	-	
2	5.49(1H, d, *J*=11.2 Hz),	79.1	5.48 (d, *J* = 11.2 Hz)	78.3
3	4.62(1H, d, *J*=11.2 Hz)	73.5	4.61 (d, *J* = 11.2 Hz)	72.7
4	-	193.0		192.2
5	-	164.1		164.0
6	6.11 (1H, s)	94.2	6.09 (d, *J* = 2.0 Hz)	93.5
7	-	167.3		166.6
8	5.98 (1H, s)	96.9	5.97 (d, *J* = 2.0 Hz)	96.2
9	-	166.4		165.7
10	-	103.7		102.9
1'	-	124.3		123.6
2'	-	157.2		156.4
3'	6.84 (1H, d, *J*=8.4Hz)	116.6	6.82 (1H, d, *J*=8.5Hz)	115.9
4'	7.17 (1H, t, *J*=7.6 Hz)	130.8	7.17 (1H, td, *J* =7.6, 1.5 Hz)	130.3
5'	6.88 (1H, d, *J*=7.6 Hz)	120.5	6.88 (1H, d, *J*=7.6Hz)	119.8
6'	7.41 (1H, d, *J*=7.6Hz)	129.5	7.41 (1H, dd, *J* =7.6, 1.5 Hz)	128.8
OCH_3	3.84 (3H, s)	56.2	3.83 (3H s,)	55.5

a) Recorded at 400 MHz. *b*) 100 MHz.

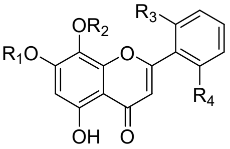

	R_1	R_2	R_3	R_4
**1**	H	H	H	H
**2**	CH_3	CH_3	H	H
**3**	CH_3	CH_3	OH	OH
**4**	H	CH_3	OH	H

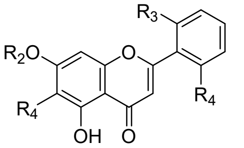

	R_1	R_2	R_3	R_4
**5**	H	H	H	H
**6**	CH_3	H	H	H
**7**	H	H	OH	OH
**8**	CH_3	CH_3	OH	OH
**9**	H	glcA	H	H

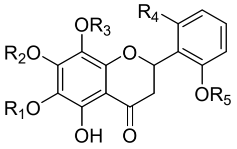

	R1	R2	R3	R4	R5
**10**	CH_3	H	H	H	H
**11**	CH_3	CH_3	CH_3	H	H

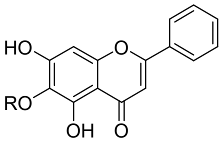

	R
**12**	H
**13**	CH_3

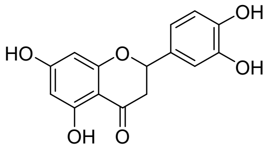

14

No.	Compounds
1	norwogonin
2	moslosooflavone
3	viscidulin II
4	scutevulin
5	baicalein
6	oroxylin A
7	2-(2,6-dihydroxyl phenyl)-5,6,7-trihydroxy-flavone
8	mosloflavone
9	baicalin
10	5,7,8-trihydroxy-6-methoxylflavone
11	alnetin
12	dihydrobaicalein
13	dihydrooroxylin A
14	5,7,30,40-tetrahydroxy flavanone

Fig. 15 Structures of isolated compounds from *S. baicalensis* in the same laboratory

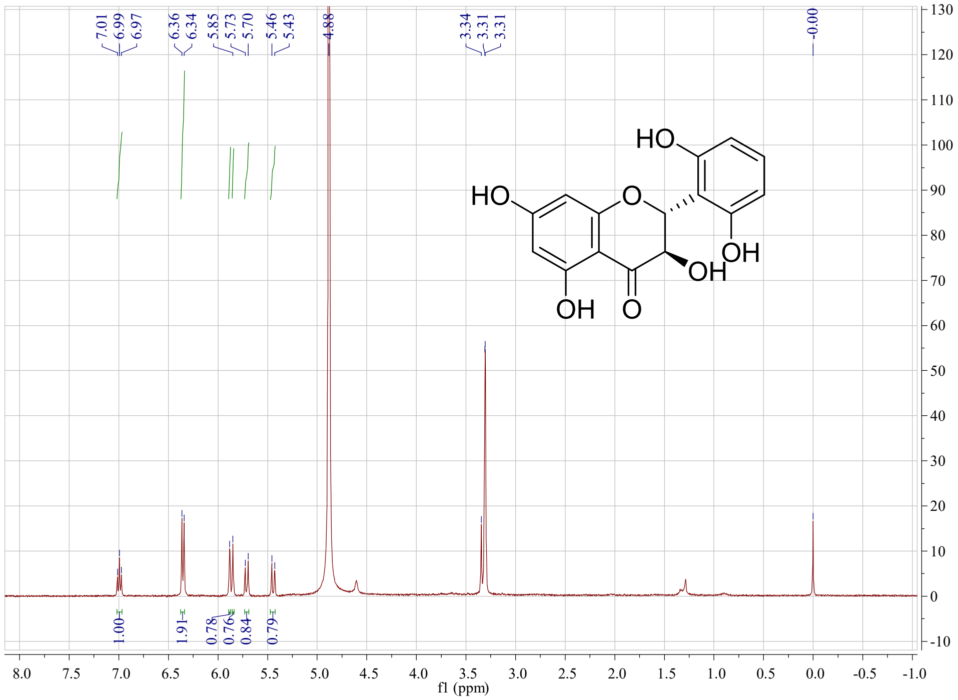

Fig. 16 ^{1}H-NMR (400Hz, CD_3OD) spectrum of compound **2**

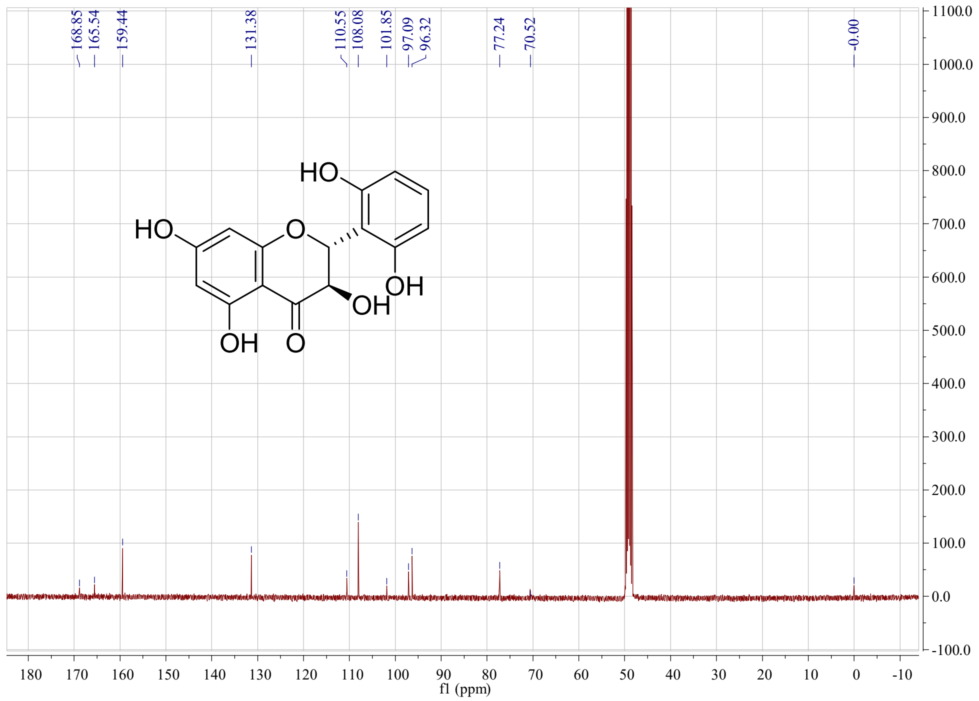

Fig. 17 ^{13}C-NMR (100Hz, CD_3OD) spectrum of compound **2**

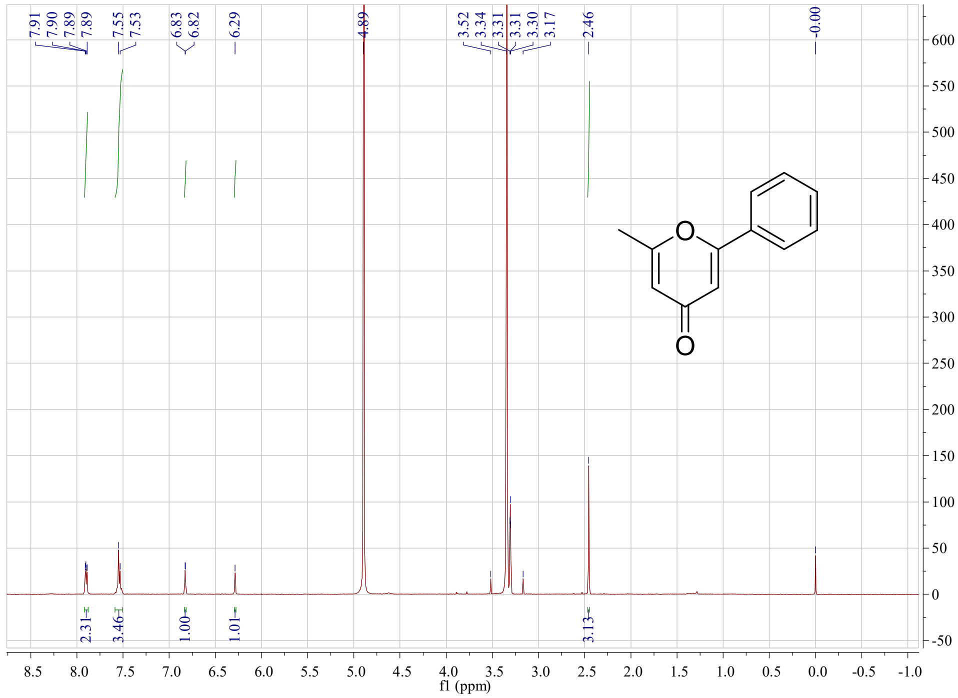

Fig. 18 ^{1}H-NMR (400Hz, CD_3OD) spectrum of compound **3**

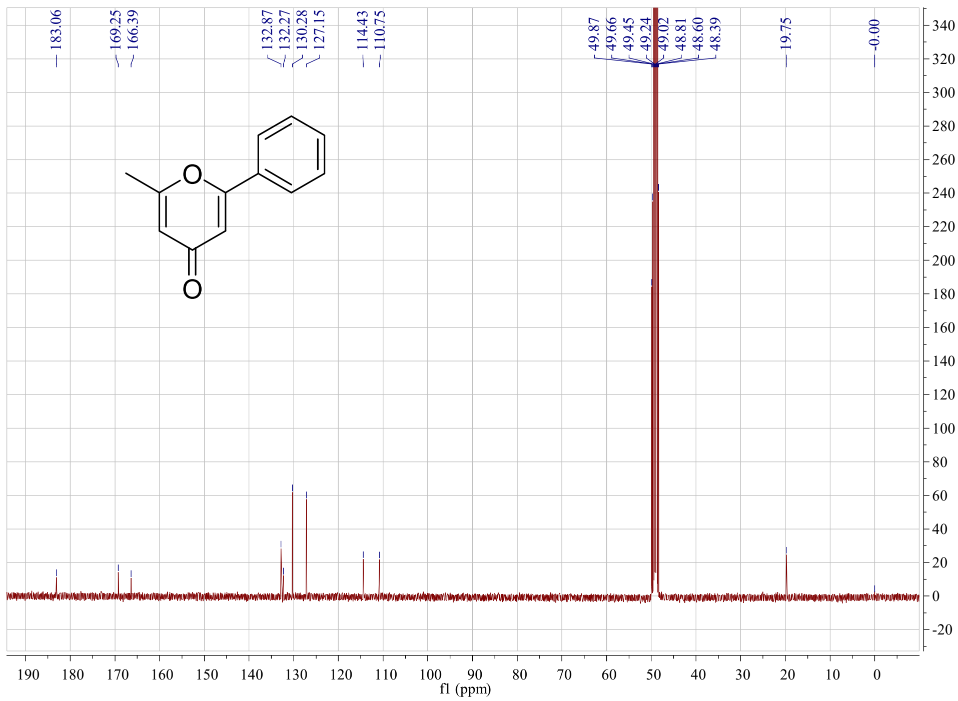

Fig. 19 ^{13}C-NMR (100Hz, CD_3OD) spectrum of compound **3**

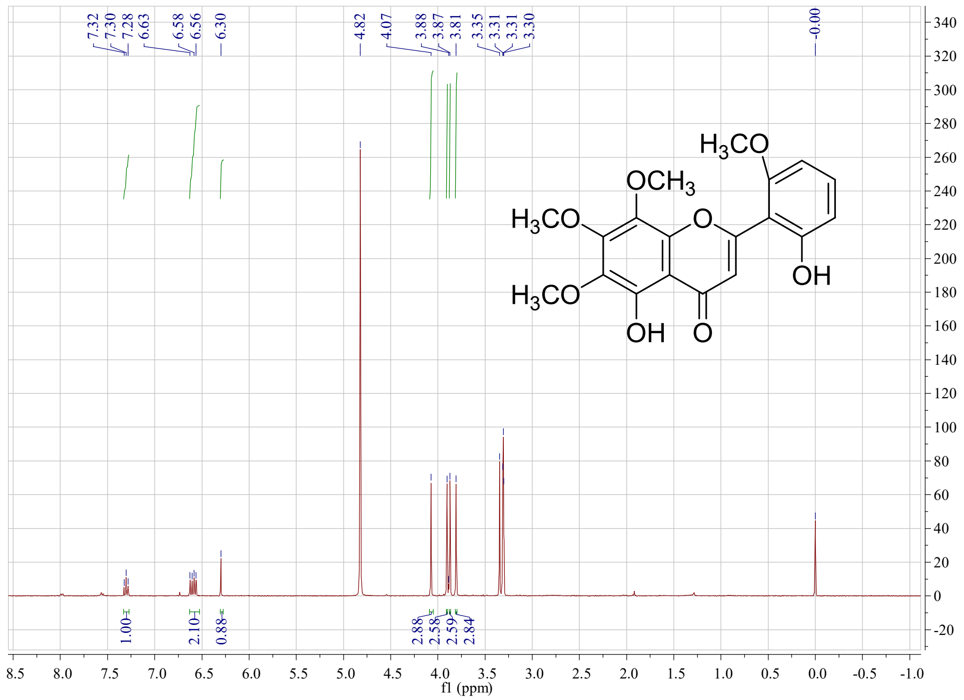

Fig. 20 ^{1}H-NMR (400Hz, CD_3OD) spectrum of compound **4**

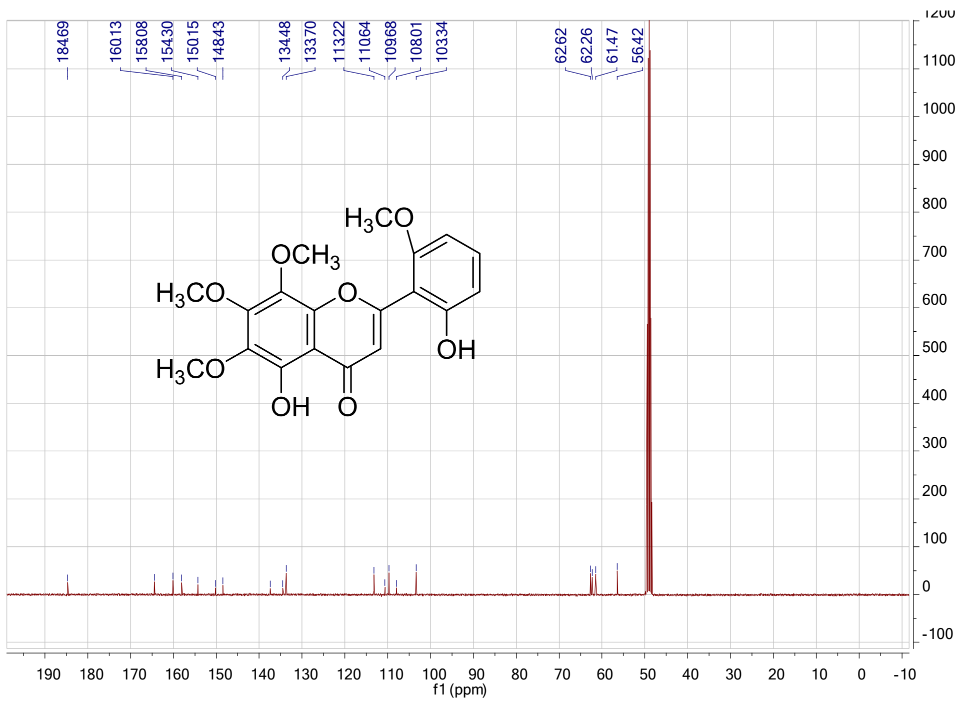

Fig. 21 ^{13}C-NMR (100Hz, CD_3OD) spectrum of compound **4**

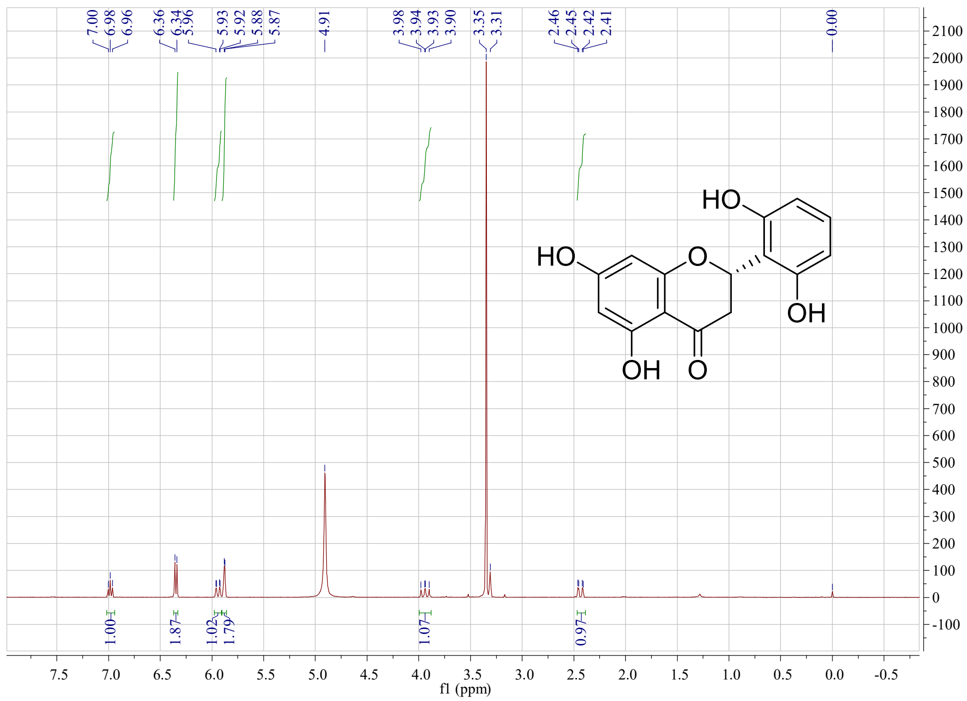

Fig. 22 ^{1}H-NMR (400Hz, CD_3OD) spectrum of compound **5**

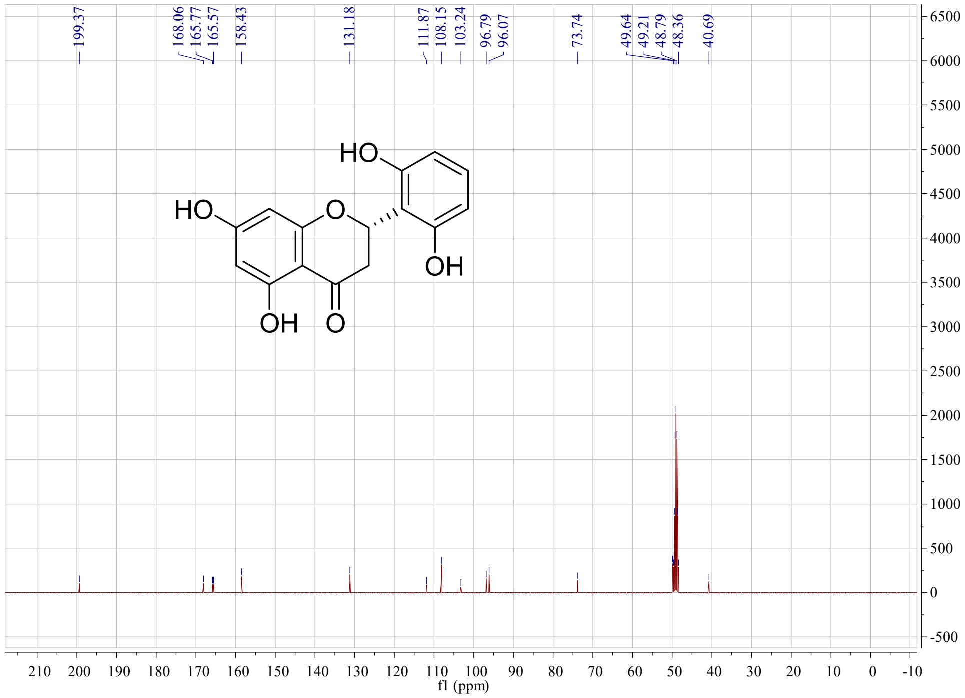

Fig. 23 ^{13}C-NMR (100Hz, CD_3OD) spectrum of compound **5**

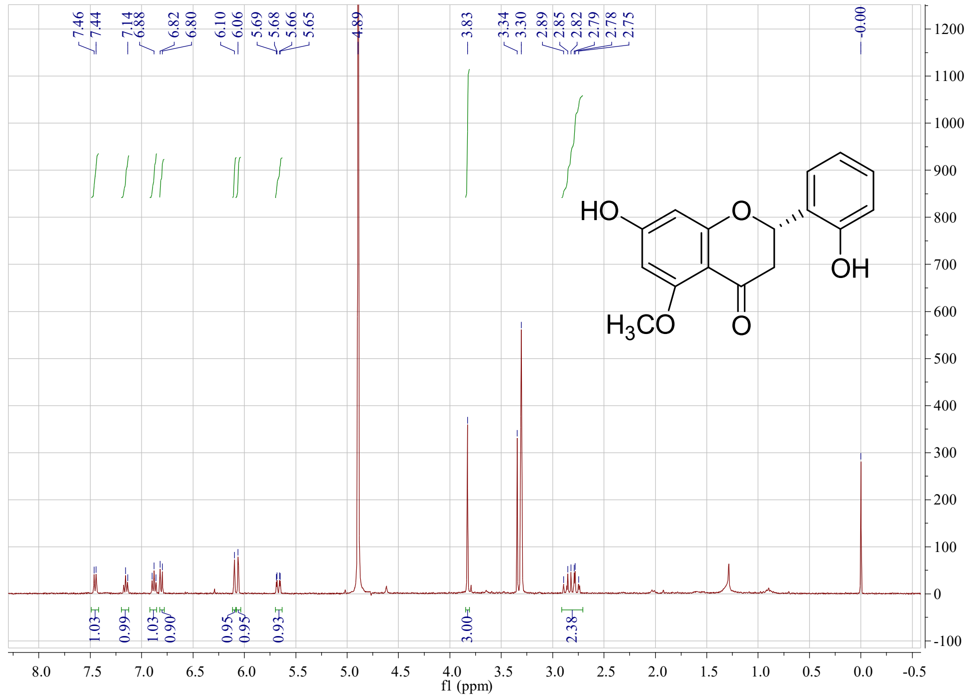

Fig. 24 ^{1}H-NMR (400Hz, CD_3OD) spectrum of compound **6**

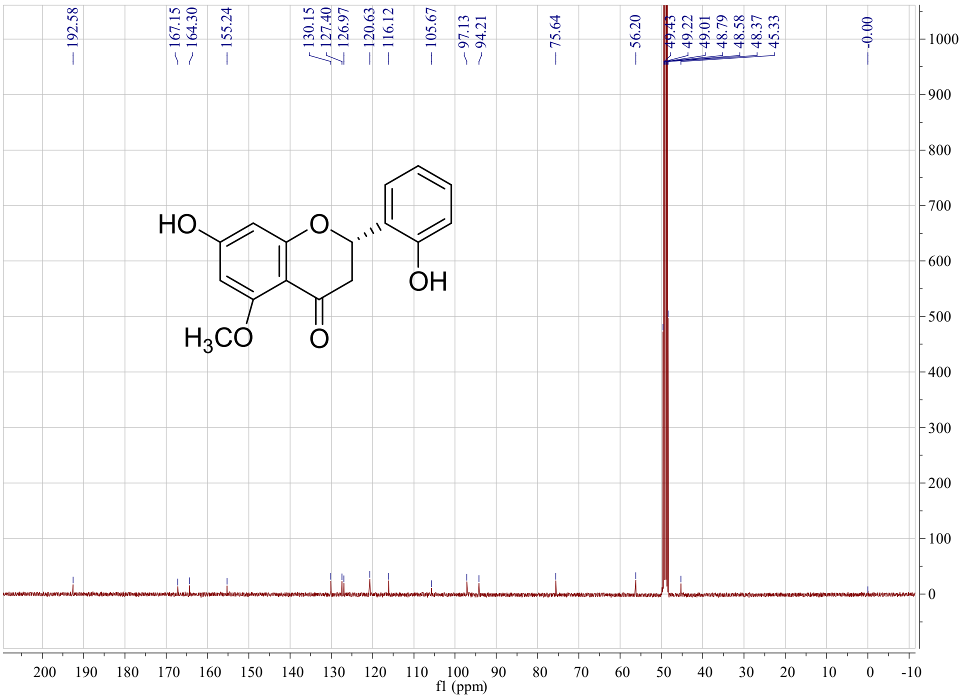

Fig. 25 ^{13}C-NMR (100Hz, CD_3OD) spectrum of compound **6**

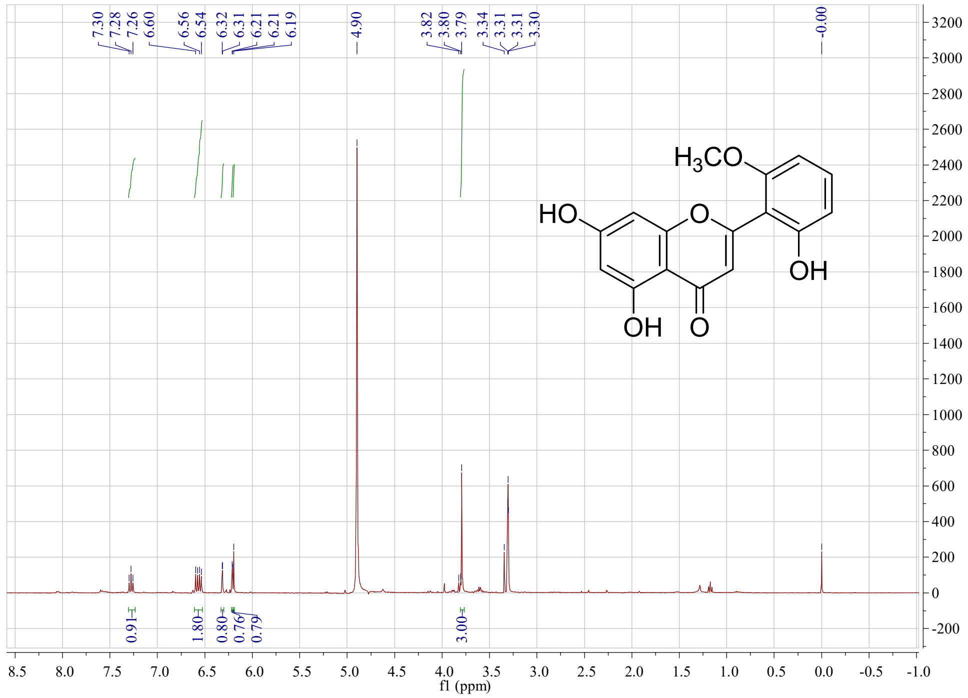

Fig. 26 ^{1}H-NMR (400Hz, CD_3OD) spectrum of compound **7**

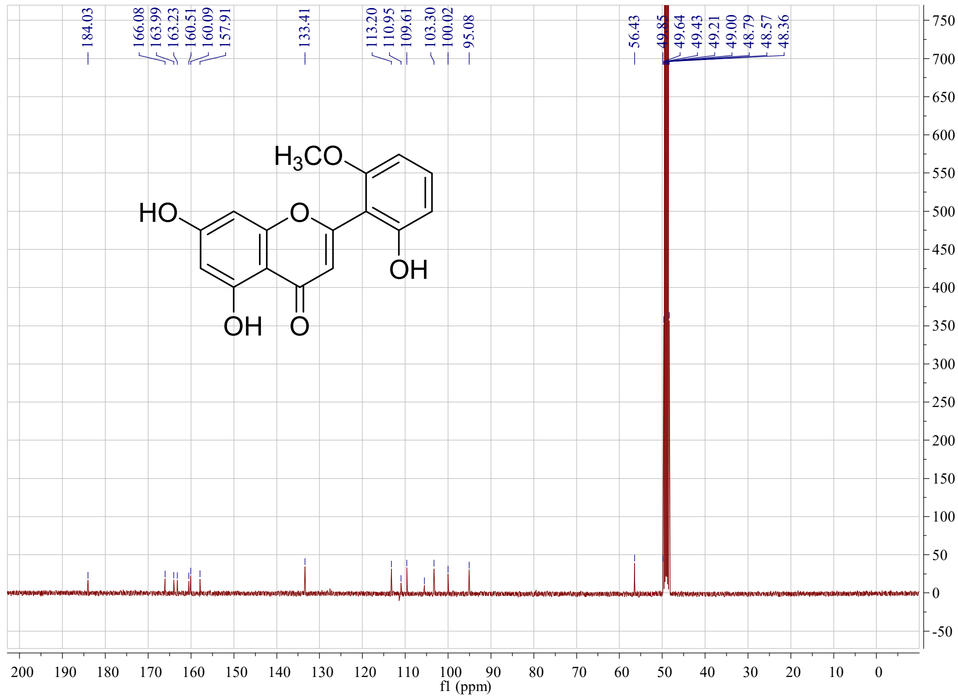

Fig. 27 ^{13}C-NMR (100Hz, CD_3OD) spectrum of compound **7**

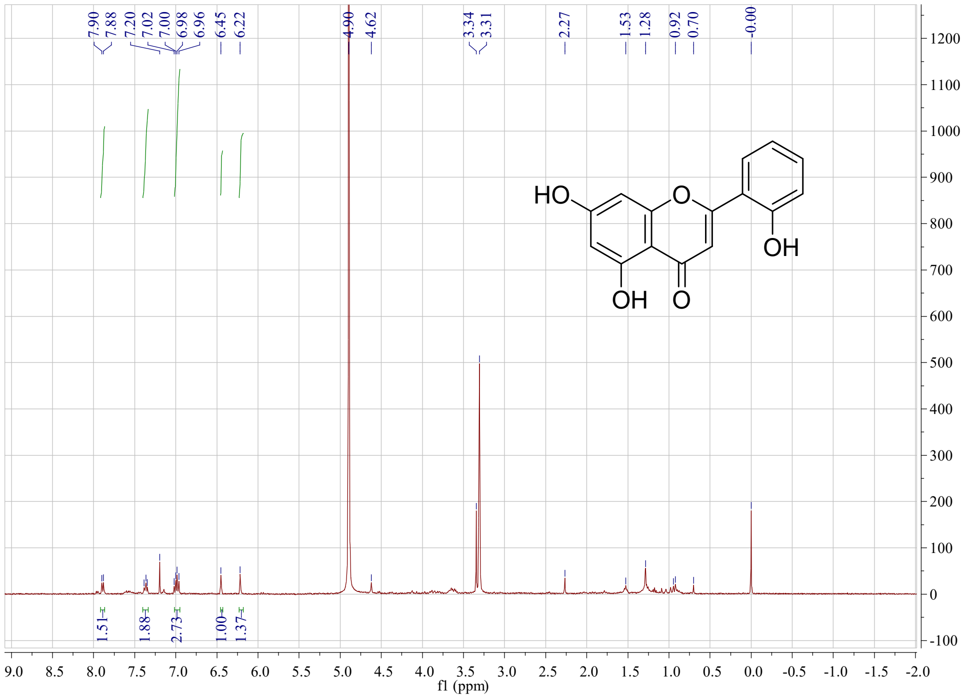

Fig. 28 ^{1}H-NMR (400Hz, CD_3OD) spectrum of compound **8**

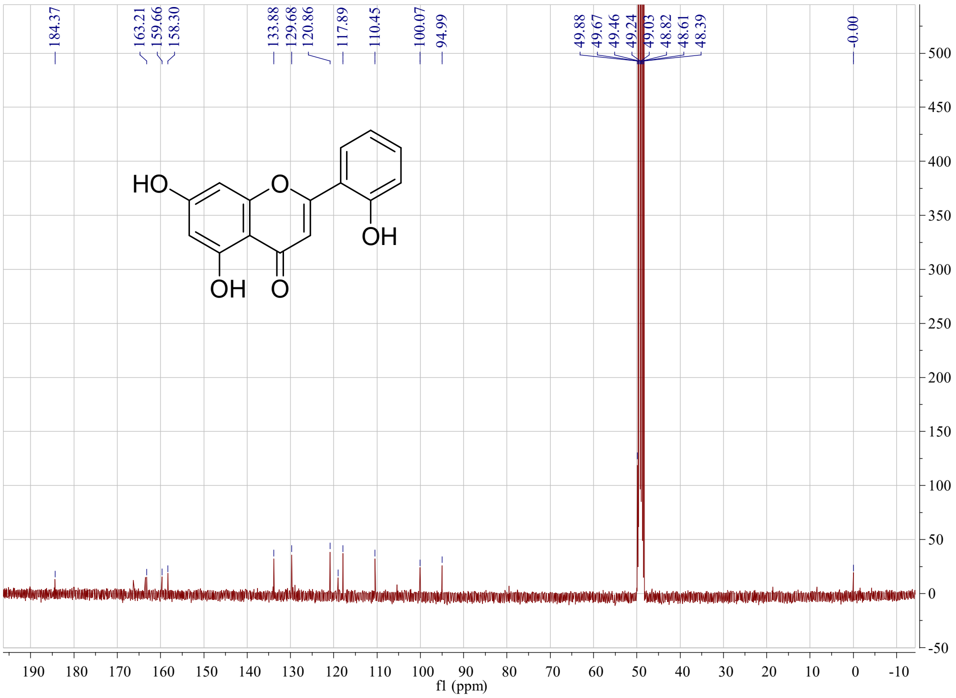

Fig. 29 ^{13}C-NMR (100Hz, CD_3OD) spectrum of compound **8**

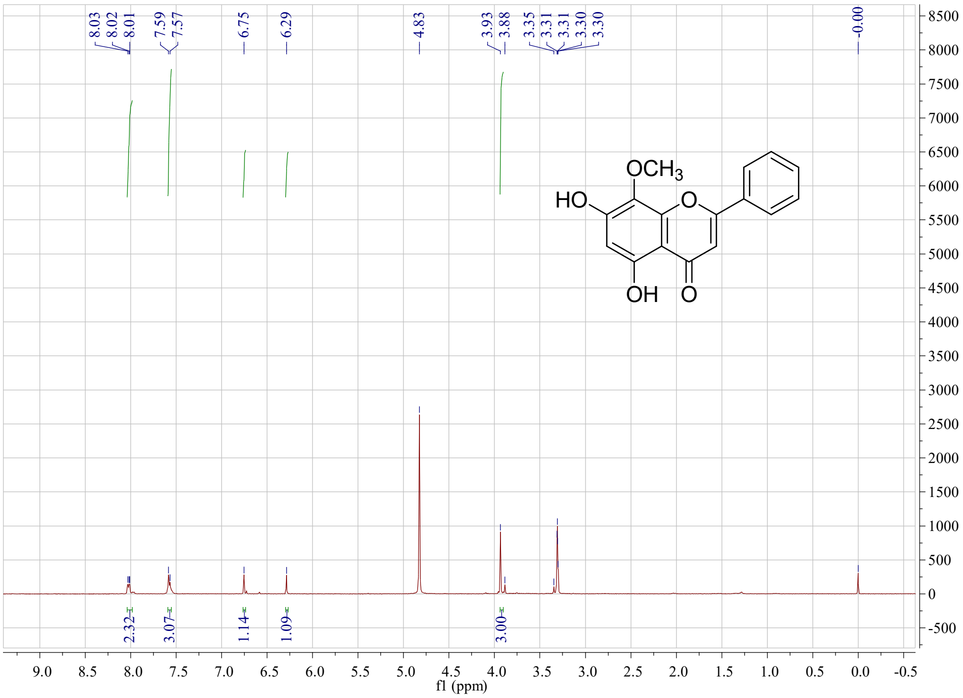

Fig. 30 ^{1}H-NMR (400Hz, CD_3OD) spectrum of compound **9**

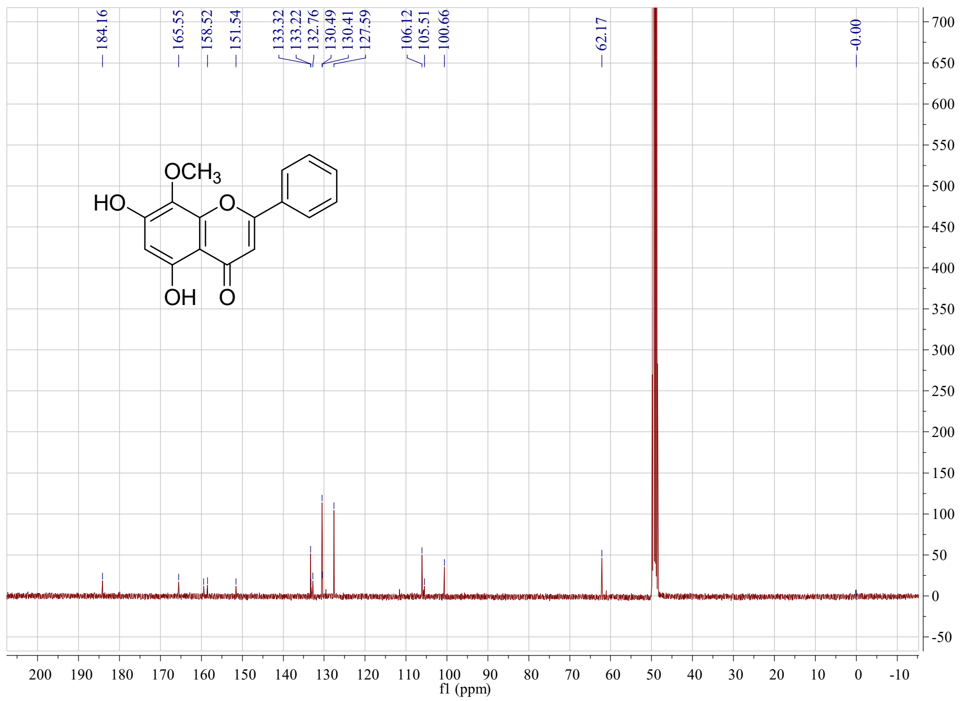

Fig. 31 ^{13}C-NMR (100Hz, CD_3OD) spectrum of compound **9**

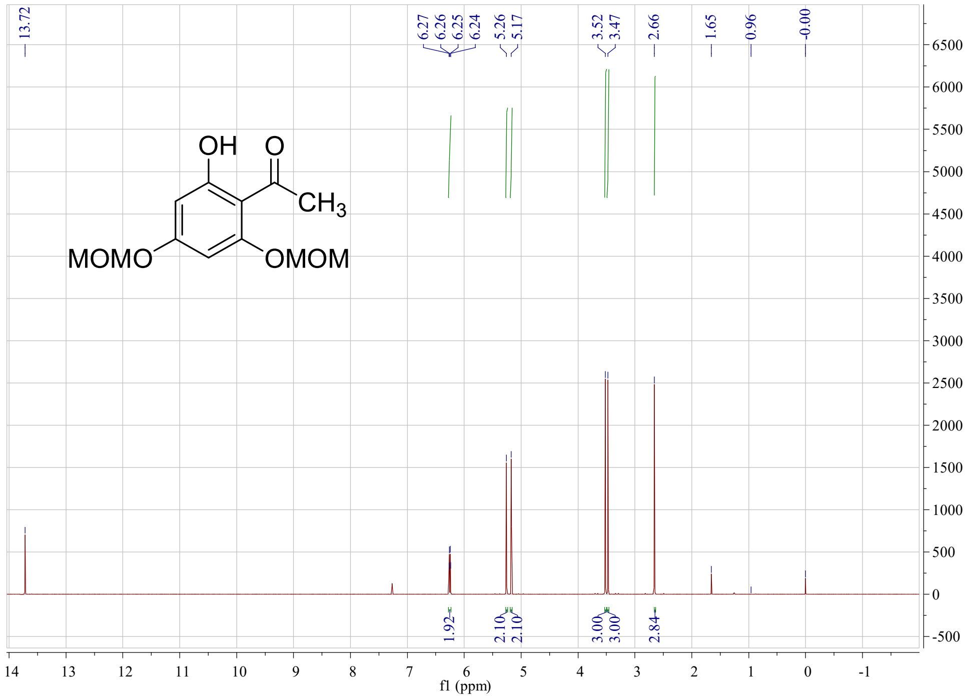

Fig. 32 ^{1}H-NMR (400Hz, CD_3OD) spectrum of compound **11**

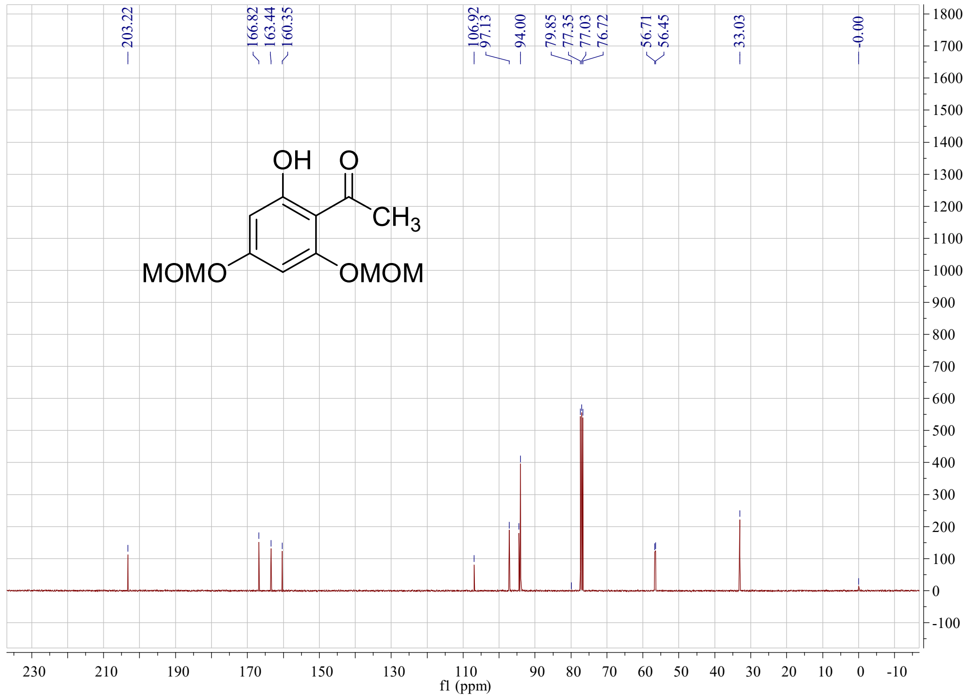

Fig. 33 ^{13}C-NMR (100Hz, CD_3OD) spectrum of compound **11**

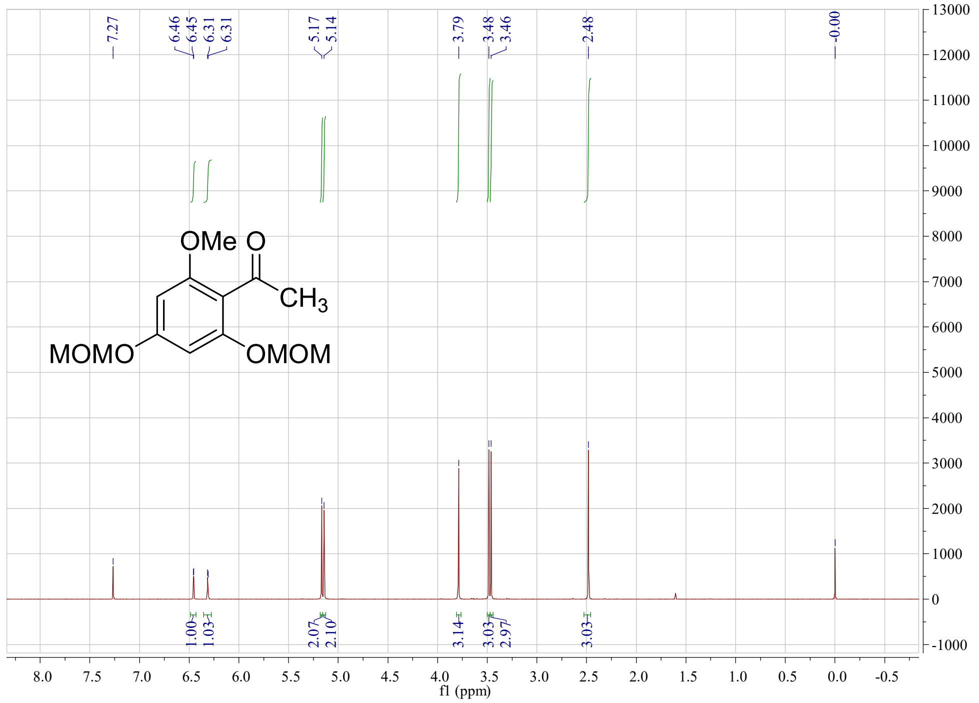

Fig. 34 ^{1}H-NMR (400Hz, CD_3OD) spectrum of compound **12**

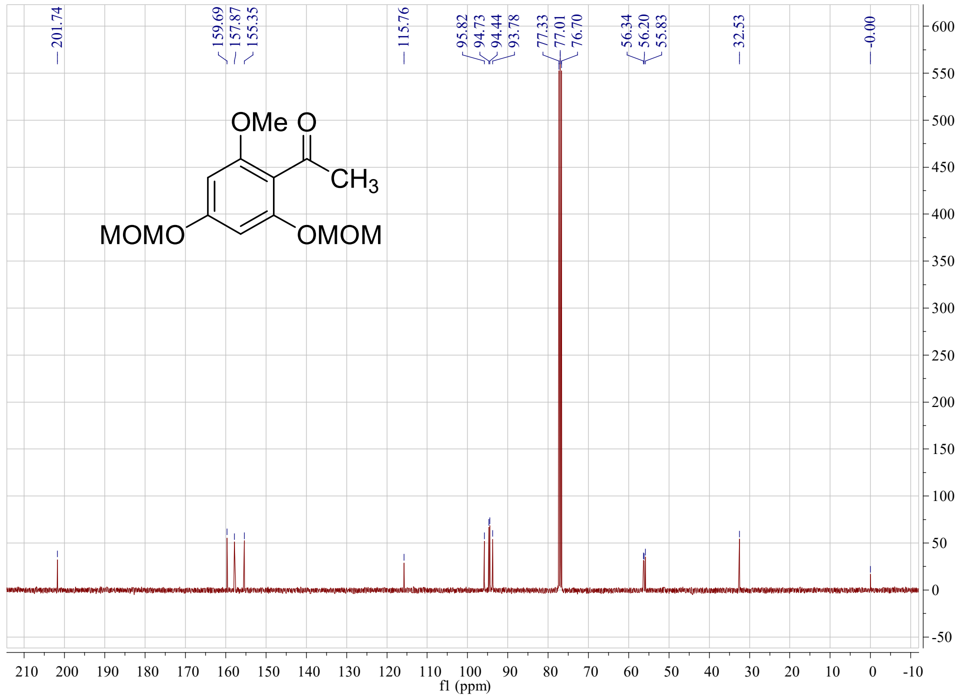

Fig. 35 ^{13}C-NMR (100Hz, CD_3OD) spectrum of compound 1**2**

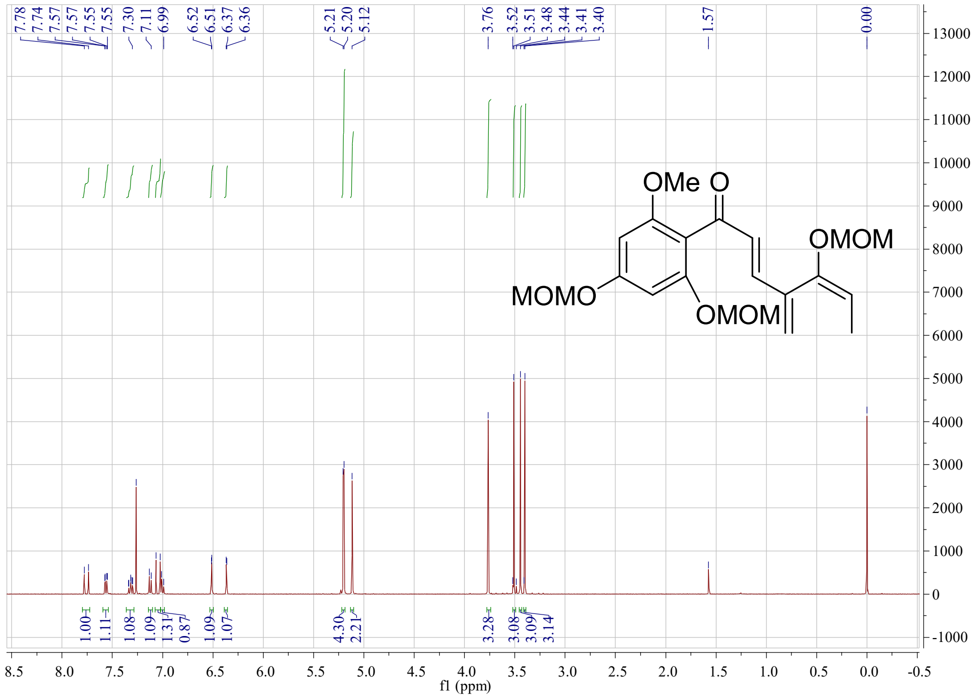

Fig. 36 ^{1}H-NMR (400Hz, CD_3OD) spectrum of compound **14**

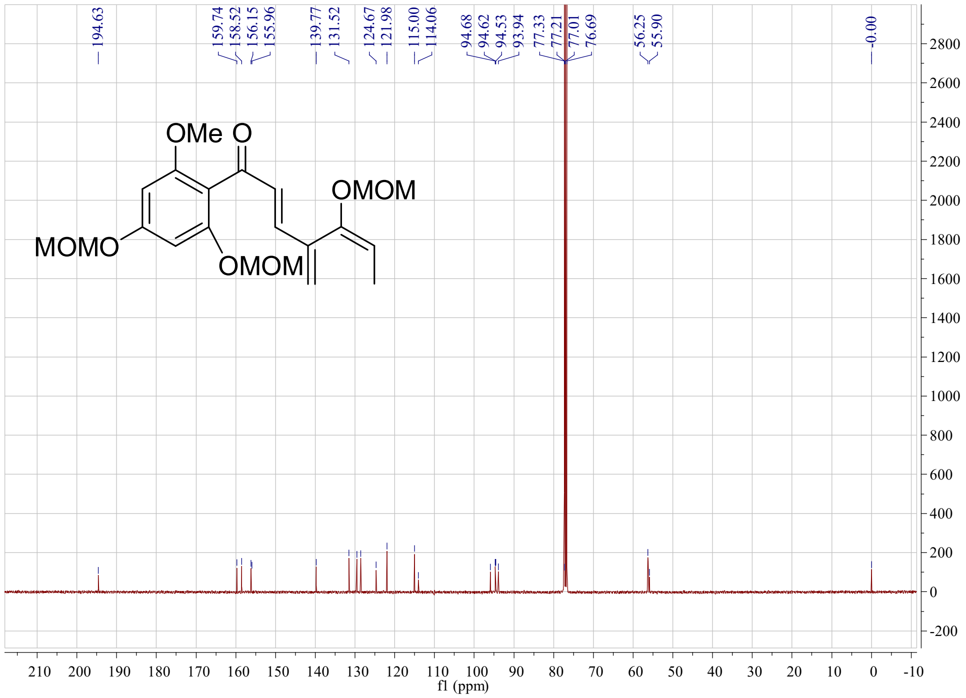

Fig. 37 ^{13}C-NMR (100Hz, CD_3OD) spectrum of compound **14**

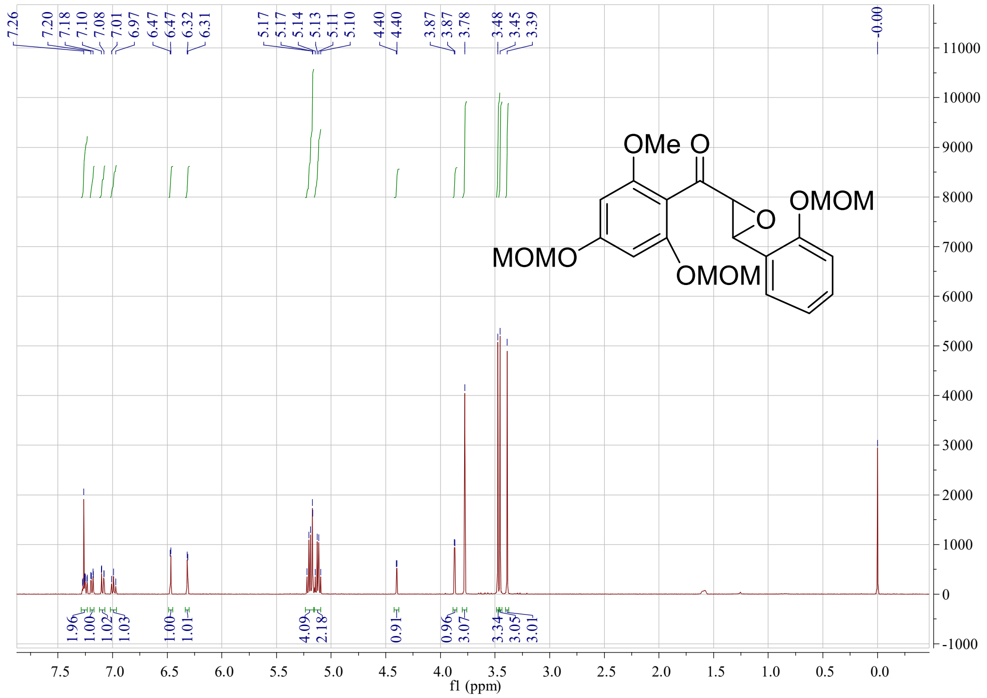

Fig. 38 ^{1}H-NMR (400Hz, CD_3OD) spectrum of compound **15**

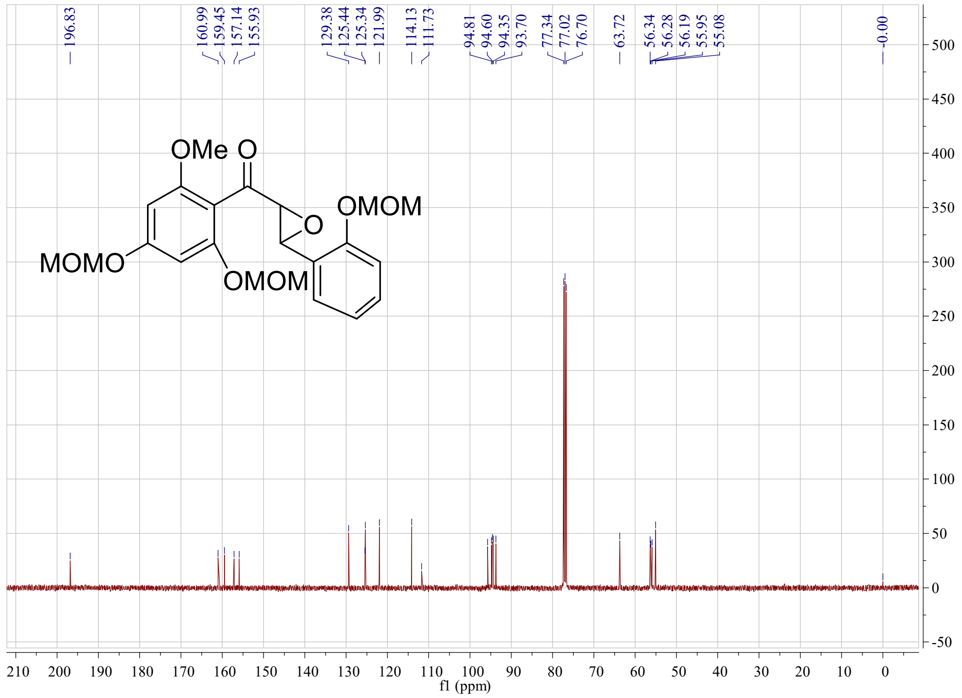

Fig. 39 ^{13}C-NMR (100Hz, CD_3OD) spectrum of compound **15**

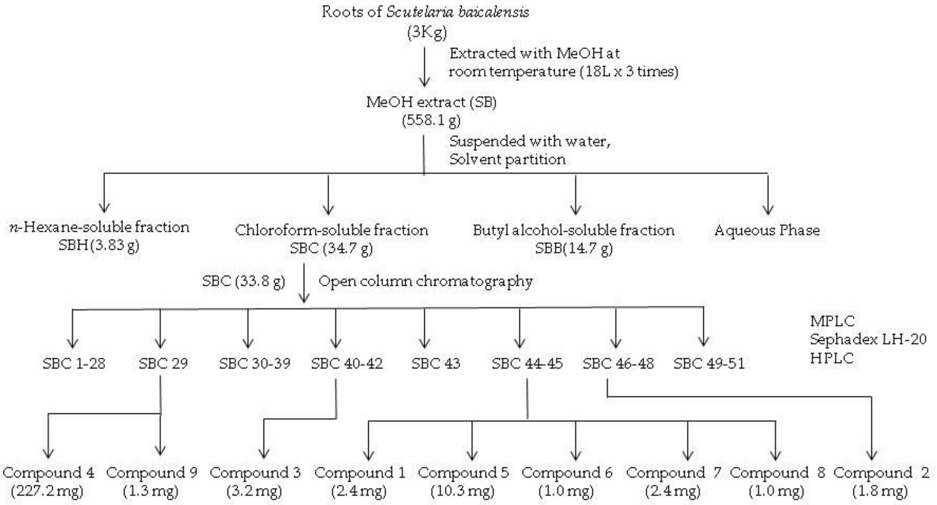

Fig. 40 Isolation scheme of *S. baicalensis*.
